# Nanocapsuled Neutrophil Extracellular Trap Scavenger Combating Chronic Infectious Bone Destruction Diseases

**DOI:** 10.1002/advs.202411274

**Published:** 2025-01-17

**Authors:** Siying Tao, Yingming Yang, Chenzhou Wu, Jiaojiao Yang, Ziyou Wang, Fangjie Zhou, Kunneng Liang, Yi Deng, Jianshu Li, Jiyao Li

**Affiliations:** ^1^ State Key Laboratory of Oral Diseases, National Center for Stomatology, National Clinical Research Center for Oral Diseases, Department of Cariology and Endodontics, West China Hospital of Stomatology Sichuan University Chengdu 610041 China; ^2^ State Key Laboratory of Oral Diseases, National Center for Stomatology, National Clinical Research Center for Oral Diseases, Department of Head and Neck Oncology West China Hospital of Stomatology Sichuan University Chengdu 610041 China; ^3^ State Key Laboratory of Oral Diseases, National Center for Stomatology, National Clinical Research Center for Oral Diseases West China Hospital of Stomatology Sichuan University Chengdu 610041 China; ^4^ School of Chemical Engineering Sichuan University Chengdu 610065 China; ^5^ College of Polymer Science and Engineering State Key Laboratory of Polymer Materials Engineering Sichuan University Chengdu 610065 China

**Keywords:** black phosphorus, neutrophil extracellular trap, periodontitis, tannic acid

## Abstract

Chronic infectious bone destruction diseases, such as periodontitis, pose a significant global health challenge. Repairing the bone loss caused by these chronic infections remains challenging. In addition to pathogen removal, regulating host immunity is imperative. The retention of neutrophil extracellular traps (NETs) in chronic infectious niches is found to be a barrier to inflammation resolution. However, whether ruining the existing NETs within the local infectious bone lesions can contribute to inflammation resolve and bone repair remains understudied. Herein, a nanocapsuled delivery system that scavenges NETs dual‐responsively to near‐infrared light as a switch and to NETs themselves as a microenvironment sensor is designed. Besides, the photothermal and photodynamic effects endow the nanocapsules with antibacterial properties. Together with the ability to clear NETs, these features facilitate the restoration of the normal host response. The immunocorrective properties and inherent pro‐osteogenic effects finally promote local bone repair. Together, the NET scavenging nanocapsules address the challenge of impaired bone repair in chronic infections due to biased host response caused by excessive NETs. This study provides new concepts and strategies for repairing bone destruction attributable to chronic infections via correcting biased host responses in chronic infectious diseases.

## Graphical abstract

Dual‐responsive nanocapsules exhibit antibacterial properties and can scavenge neutrophil extracellular traps (NETs) within infectious niches, crucial for restoring normal host responses. Their immunocorrective properties and inherent pro‐osteogenic effects facilitate local bone repair. Collectively, the study resolves the issue of impaired bone repair in chronic infections due to biased host response caused by excessive NETs.

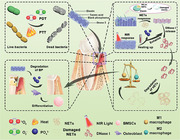

## 1 Introduction

Chronic infectious bone destruction is a major global health concern. Periodontitis, for example, has a high prevalence of 45–50% in the world's adult population and is the sixth most common disease in humans. Periodontitis is a disease characterized by immunoinflammatory destruction of the periodontal tissues, resulting in the detachment of soft tissue and the resorption of alveolar bone due to the activity of periodontal pathogenic microorganisms.^[^ ^1^, ^2^ ^]^ The repair of bone destruction caused by chronic infections remains rather intractable. The interactions between the bacteria and the host determine the outcomes of bone tissue in chronic infections.

In addition to removing pathogenic bacteria, it is imperative to regulate host immunity during chronic infections.^[^ ^3^ ^]^ Neutrophil extracellular traps (NETs) are web‐like structures developed by neutrophils in response to infections. Through a type of cell death process called NETosis, neutrophils release sticky DNA scaffolds containing proteases or peptides into the extracellular spaces.^[^ ^4^ ^]^ Due to the large web‐like structures of NETs, it is difficult for scavenger cells to engulf and clear them. Moreover, the DNA components within NETs are highly oxidative, contributing to severe vascular and tissue damage.^[^ ^5^ ^]^ Excessive accumulation of NETs leads to autoimmune clearance dysfunction, which impedes bone tissue healing.^[^ ^6^, ^7^, ^8^, ^9^ ^]^ In NETs, DNA is a scaffold dotted with granule proteins, among which neutrophil elastase is of utmost priority.^[^ ^7^ ^]^ Neutrophil elastase is one of the most abundant proteases attached to NETs and can act as a detection target. Elastin is a specific substrate for neutrophil elastase.^[^ ^10^ ^]^

Studies have shown that the accumulation and continual activation of neutrophils and the subsequent NETs production are closely associated with the development of chronic infectious bone destruction, including periodontitis.^[^ ^11^, ^12^, ^13^ ^]^ NETs cause an excessive inflammatory response and persistent inflammatory conditions in periodontitis, thereby triggering oral mucosal immunopathology.^[^ ^14^, ^15^, ^16^ ^]^ Several therapeutic agents targeting NETs, such as DNase I and protein‐arginine deaminase 4 inhibitors, have demonstrated efficacy in experimental cancer treatments. DNase I, an endonuclease that cleaves DNA, has shown promise as a candidate for disrupting NETs in cancer patients. Notably, DNase I has already received approval from the U.S. Food and Drug Administration for the treatment of cystic fibrosis.^[^ ^17^ ^]^ However, whether disrupting the existing NETs within the local infectious bone lesions can contribute to inflammation resolution and bone repair remains unexplored.

**Figure 1:**
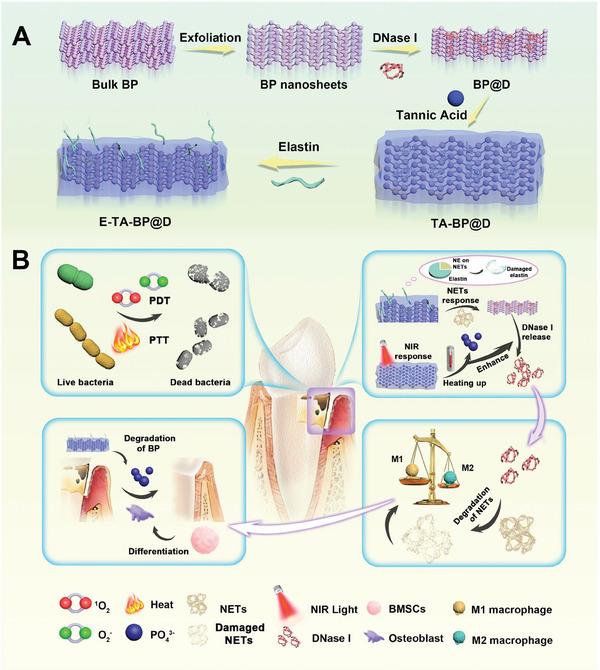
Schematic illustration of the study. A) Schematic illustration of the preparation of E‐TA‐BP@D. B) Schematic demonstration of the mechanisms of E‐TA‐BP@D for chronic infectious bone destruction diseases treatment (using periodontitis as an example). When E‐TA‐BP@D is applied with NIR light irradiation, the photothermal (PTT) and photodynamic (PDT) effects kill the bacteria in the infection niche. BP degradation is enhanced shortly in response to NIR light, releasing DNase I rapidly to scavenge excessive NETs in the infection niche. When without NIR light irradiation in the later stage, the degradation of E at the outer edge of delivery vehicle accelerates the release of DNase I in response to neutrophil elastase (NE) on NETs, realizing targeted scavenging of NETs. The dual‐responsive scavenging of NETs further promotes the transformation of macrophage M1 type to M2 type to block the pathological damage caused by NETs. Finally, with the co‐effect of the BMSCs osteogenesis differentiation promoted by polarization‐transformed macrophages and the high phosphorus ion concentration microenvironment created by BP degradation, the osteogenesis process of bone resorption is enhanced, repairing bone defect.

Herein, we design a localized controlled‐release photoreactive material to regulate the bacterial–host interactions and correct the biased host response arising from overactivated NETs. A nanocapsuled delivery system consisting of a black phosphorus (BP) carrier loaded with DNase I (D), peripherally modified with tannic acid (TA) and elastin (E), namely E‐TA‐BP@D, is devised (**Figure** **1**A), to achieve the controlled release of DNase I in a near‐infrared (NIR) light and NETs double‐responsive manner. BP can be a good carrier of DNase I.^[^ ^18^, ^19^ ^]^ In addition, BP has excellent optical properties; under NIR light irradiation, increased temperature and accelerated degradation of BP simultaneously accelerate drug release to realize NIR light‐responsive drug release.^[^ ^20^ ^]^ The photothermal and photodynamic effects of BP can kill bacteria in an infection niche.^[^ ^21^, ^22^ ^]^ Moreover, BP degradation products provide phosphorus sources for apatite regeneration, promote biomineralization, and facilitate osteogenesis.^[^ ^23^ ^]^ Peripheral modification of nanocapsules with elastin can endow them with NET‐targeting properties. The degradation of elastin by neutrophil elastase can accelerate the release of DNase I, fulfilling NETs‐response. Catechol‐rich TA improves the stability of BP and assists in elastin modification.^[^ ^24^, ^25^, ^26^ ^]^

Our results show that the nanocapsules realize NETs degradation and acquire antibacterial ability under NIR light irradiation, which is beneficial for restoring the normal host response. These processes are associated with macrophage polarization. The immunomodulatory effects and material degradation products together boost the osteogenic differentiation of bone marrow mesenchymal stem cells (BMSCs) within the inflammatory niche and further promote bone repair (Figure 1B). We address the issue of impaired bone repair in chronic infections due to biased host response caused by the accumulation of NETs. Our study provides new concepts and strategies for repairing bone destruction attributable to chronic infections via correcting biased host responses in chronic infectious diseases.

## 2 Results

### 2.1 Synthesis and Characterization of E‐TA‐BP@D

**Figure 2:**
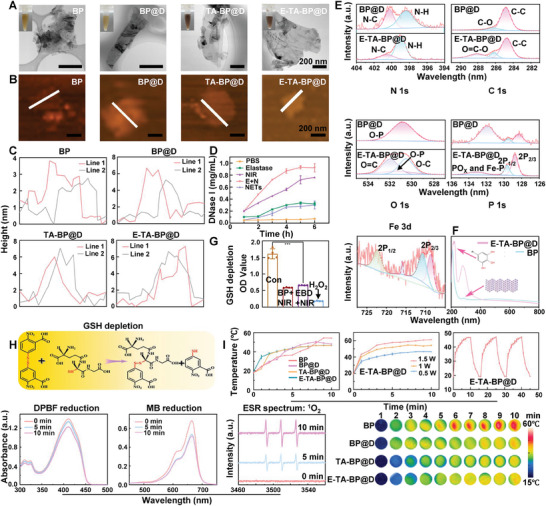
Characterization, photothermal, and photodynamic performances. A) TEM images of BP, BP@D, TA‐BP@D, and E‐TA‐BP@D. B) AFM images of BP, BP@D, TA‐BP@D, and E‐TA‐BP@D. C) Height profiles of BP, BP@D, TA‐BP@D, and E‐TA‐BP@D. D) DNase I release profiles under different stimuli. E+N: Elastase+NIR. E) High‐resolution N 1s, C 1s, O 1s, P 1s, and Fe 3d XPS patterns. F) UV–vis DRS patterns of BP and E‐TA‐BP@D. G,H) Photodynamic performance of E‐TA‐BP@D. Data are presented as mean ± SD (*n* = 3) and analyzed using a one‐way ANOVA,***p < 0.001. Con: Control and EBD+NIR: E‐TA‐BP@D+NIR. I) Photothermal performance of BP‐based nanocapsules.

BP nanosheets, BP@D, TA‐BP@D, and E‐TA‐BP@D were prepared sequentially, as detailed in the Experimental Section. The BP and BP@D suspensions displayed a brownish–yellow appearance, whereas TA‐BP@D and E‐TA‐BP@D displayed a brownish–black appearance due to TA modification (**Figure** **2**A). BP showed a typical single‐layered nanosheet morphology with a long diameter of ≈200–400 nm in transmission electron microscopy (TEM) images. Field emission scanning electron microscopy (FE‐SEM) images indicated that the BP nanosheets were fully covered with protein aggregation particles of DNase I, which is consistent with the TEM findings. This occurred because of the large number of negatively charged termination groups on the BP surface, facilitating the adsorption of DNase I. Both the TEM and FE‐SEM images of E‐TA‐BP@D showed that the elastase agglomeration particles adhered to the outermost layer of the BP‐based nanocapsules (Figure 2A; Figure S1, Supporting Information). Atomic force microscopy (AFM) results indicated that all four groups displayed typical 2D nanosheet characteristics (Figure 2B). The nanosheet heights in the four groups were ≈3, 6, 7, and 8 nm (Figure 2C). The thickness of the BP was similar to the theoretical value reported for a monolayer.^[^ ^27^ ^]^ With the loading of DNase I and grafting of TA and E, the thickness of the BP‐based nanocapsules gradually increased.^[^ ^28^ ^]^ The load efficiency of DNase I was calculated to be 36.01 ± 6.43%. DNase I release curves are shown in Figure 2D. DNase I was released in a dual‐response manner to NETs and NIR light. NETs, elastase, and NIR light irradiation significantly promoted DNase I release.

The X‐ray photoelectron spectroscopy (XPS) spectra of BP@D and E‐TA‐BP@D revealed distinct P, C, O, and N peaks (Figure S2, Supporting Information). The high‐resolution N 1s, C 1s, O 1s, P 1s, and Fe 3d XPS patterns are shown in Figure 2E. In the N 1s and O 1s patterns, peaks at 400.6 and 531.4 eV could be observed, attributed to C─N and C═O bonds and indicating the presence of amide bonds in both BP@D and E‐TA‐BP@D. A semi‐quantitative analysis of the C═O bond revealed that E‐TA‐BP@D contained more amide bonds than BP@D, suggesting the loading of E (Figure S3, Supporting Information). The P 1s spectrum showed a peak at 133 eV, attributed to the P─O bond (Figure 2E). Semi‐quantitative analysis further demonstrated that BP@D contained more P─O bonds, likely because TA slowed BP oxidation in E‐TA‐BP@D (Figure S3, Supporting Information). The Fe 3d spectrum demonstrated that the Fe peak in E‐TA‐BP@D confirmed the formation of TA. Dynamic light scattering (DLS) and zeta potential measurements showed that BP and BP@D exhibited particle sizes of 500–600 nm, larger than those obtained from TEM, suggesting a tendency for aggregation in water (Figures S4 and S5, Supporting Information). However, TA‐BP@D and E‐TA‐BP@D exhibited particle sizes of 250 nm due to improved dispersibility of the BP‐based nanosheets by TA (Figure S4, Supporting Information). Zeta potential results indicated that with the loading of DNase I into BP, the potential increased from −20 to −5 mV. Nevertheless, with the coating of TA, the potential restored to −20 mV (Figure S5, Supporting Information). The reduced degradation rate of BP by TA was further confirmed through consecutive DLS analyses for 7 d as the particle size of E‐TA‐BP@D decreased significantly more slowly in either air or water (Figure S6, Supporting Information). UV–vis diffuse reflection spectroscopy (UV–vis DRS) results showed no significant difference in absorption at 800 nm between E‐TA‐BP@D and pure BP, indicating that the grafting of protein and polyphenol layers did not affect the absorbance. A prominent absorption peak at 200 nm, indicative of TA,^[^ ^29^ ^]^ was observed for E‐TA‐BP@D (Figure 2F). These findings indicated the successful preparation of E‐TA‐BP@D with good dispersibility, stability, and a dual‐responsive ability to release DNase I triggered by both NIR light and the presence of NETs themselves.

### 2.2 E‐TA‐BP@D is Shown as a Superior Photodynamic and Photothermal Platform

Glutathione (GSH), a tripeptide containing mercaptan groups, plays a crucial role in cellular antioxidant systems by oxidizing organic mercaptans (R‐SH) to disulfides (R–S–S–R).^[^ ^30^ ^]^ The BP+NIR and E‐TA‐BP@D+NIR groups showed significant GSH consumption, indicating the excellent photodynamic effects of E‐TA‐BP@D under NIR light illumination (Figure 2G,H). The generation of ·O_2_^−^, ^1^O_2_, and ·OH was assessed using methylene blue (MB) and 1,3‐diphenylisobenzofuran (DPBF). Compared to the untreated MB or DPBF (Figures S7 and S8, Supporting Information), a continuous decrease in absorption could be observed in MB or DPBF under NIR laser irradiation due to the generation of ·OH, ·O_2_^−^, and ^1^O_2_ in the E‐TA‐BP@D+NIR group, indicating effective electron–hole separation and their subsequent capture of absorbed oxygen to generate reactive oxygen species (ROS). Previous studies reported that BP primarily generates ^1^O_2_ under NIR light excitation.^[^ ^27^ ^]^ Therefore, electron spin resonance (ESR) was used to further demonstrate the generation of ^1^O_2_ (Figure 2H). These results demonstrate that E‐TA‐BP@D maintained a good photodynamic effect after the loading of DNase I and the coating of TA or elastin upon 808 nm light illumination.

The photothermal properties of the BP‐based nanocapsules in water were evaluated using infrared thermal imaging. As the 808 nm NIR light continued to irradiate, the temperature of each group gradually increased. At 10 min, the temperature of the BP group was significantly higher, whereas that of the other groups showed minimal differences. E‐TA‐BP@D exhibited varying degrees of temperature increase at 808 nm NIR light with power densities of 0.5, 1.0, and 1.5 W cm^−2^, reaching 43 °C, 43 °C, and 58 °C, respectively. 1.5 W cm^−2^ was used in subsequent experiments. In addition, negligible changes were detected in the cyclic up–down temperature over the three cycles. These results confirmed the excellent photothermal stability and capability of E‐TA‐BP@D, making it an outstanding photothermal platform (Figure 2I).

### 2.3 Cytocompatible E‐TA‐BP@D Exhibits Advantageous Antibacterial Efficiency With NIR Light Irradiation

**Figure 3:**
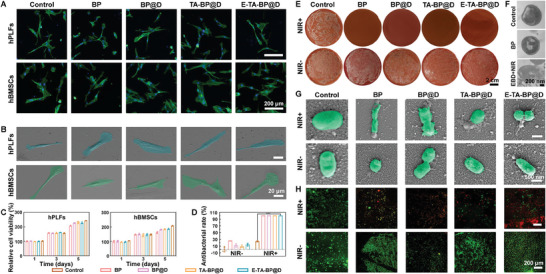
In vitro cytotoxicity and antibacterial efficiency. A) Cell structure captured by CLSM. B) Morphologies of cells in different treatment groups captured by SEM. C) Cytotoxicity assessment by CCK‐8 assay. D) Antibacterial rates calculated from the plate counting results (*P. gingivalis*). Data are presented as mean ± SD (*n* = 3) and analyzed using a one‐way ANOVA, ***p < 0.001. E) Typical bacterial plate images in different treatment groups (*P. gingivalis*). F) TEM pictures of *P. gingivalis* in different treatment groups. EBD+NIR: E‐TA‐BP@D+NIR. G) Morphologies of *P. gingivalis* in different treatment groups captured by SEM. H) Representative live/dead bacterial staining images (*P. gingivalis*).

The excellent cellular compatibility serves as the foundation for utilizing E‐TA‐BP@D for disease treatment. To assess the cytotoxicity, five representative cell types, human periodontal ligament fibroblasts (hPLFs), human BMSCs (hBMSCs), human oral keratinocyte (HOK) cell line, mouse fibroblast cell line (L929), and mouse BMSCs (mBMSCs), were employed. Confocal laser scanning microscopy (CLSM) images show that the cells exhibited well‐defined and clear nuclei, along with a normal cytoskeletal structure (**Figure** **3**A; Figures S9–S11A, Supporting Information). SEM results demonstrated that five cell types exhibited and maintained a normal morphology (Figure 3B; Figures S9–S11B, Supporting Information). The cell counting kit‐8 (CCK‐8) assay revealed no significant difference in proliferative activity between the experimental and Control groups on days 1 and 3. On day 5, the proliferative activity of the Control group surpassed that of the other groups. The proliferative activity of pure BP was lower than that of other BP‐based nanocapsules. These findings indicated that cells were still capable of sustained proliferation with BP‐based nanocapsules, and that BP‐based nanocapsules showed lower cytotoxicity than BP alone (Figure 3C; Figures S9–S11C, Supporting Information). Thus, it can be concluded that E‐TA‐BP@D exhibited a favorable compatibility with cellular systems.

BP‐based nanocapsules with NIR laser irradiation showed effective antibacterial capacity upon 808 nm laser irradiation, and the antibacterial rates calculated from the plate counting results of *Porphyromonas gingivalis* (*P. gingivalis*, the primary pathogen in chronic periodontitis^[^ ^11^ ^]^) and *Enterococcus faecalis* (*E. faecalis*, the primary pathogen in refractory periapical periodontitis^[^ ^31^ ^]^) in the E‐TA‐BP@D+NIR group were 99.95 ± 0.03% and 99.79 ± 0.30%, respectively. Viable bacteria showed green fluorescence and dead bacteria showed red fluorescence in live/dead staining images. Almost all *P. gingivalis* and *E. faecalis* exhibited green fluorescence in the NIR‐ or Control groups. In contrast, significant red fluorescence was observed in the NIR+ groups. Yellow fluorescence signal could be observed after the co‐localization of red and green fluorescence. When mixed with equal intensity, the red and green fluorescence produced yellow light, which appeared as yellow. In cases where both dead and live bacteria were present at a site, the merged image would display yellow, a phenomenon that had also been observed in previously reported images.^[^ ^32^, ^33^ ^]^ Subsequently, the bactericidal mechanism of BP‐based nanocapsules was investigated using SEM and TEM. In the NIR‐ or Control groups, both *P. gingivalis* and *E. faecalis* displayed completely rod‐shaped or spherical morphology with intact cytoplasmic membranes; while, the cell membranes of bacteria in the NIR+ groups exhibited severe damage and cytoplasmic leakage, indicating remarkable antibacterial efficacy through the synergistic effect of photothermal and photodynamic strategies. The TEM analysis revealed similar changes. The Control and BP groups had intact bacterial cell membranes and clear compact cytoplasm. However, in the E‐TA‐BP@D+NIR group, the cell walls of both *P. gingivalis* and *E. faecalis* were severely damaged and incomplete, accompanied by cytoplasmic leakage. Bubble‐like structures were observed within the bacteria, and the DNA became invisible (Figure 3D–H; Figures S12–S16, Supporting Information).

### 2.4 E‐TA‐BP@D Functions as a Targeted Net Scavenger Under NIR Light Illumination

**Figure 4:**
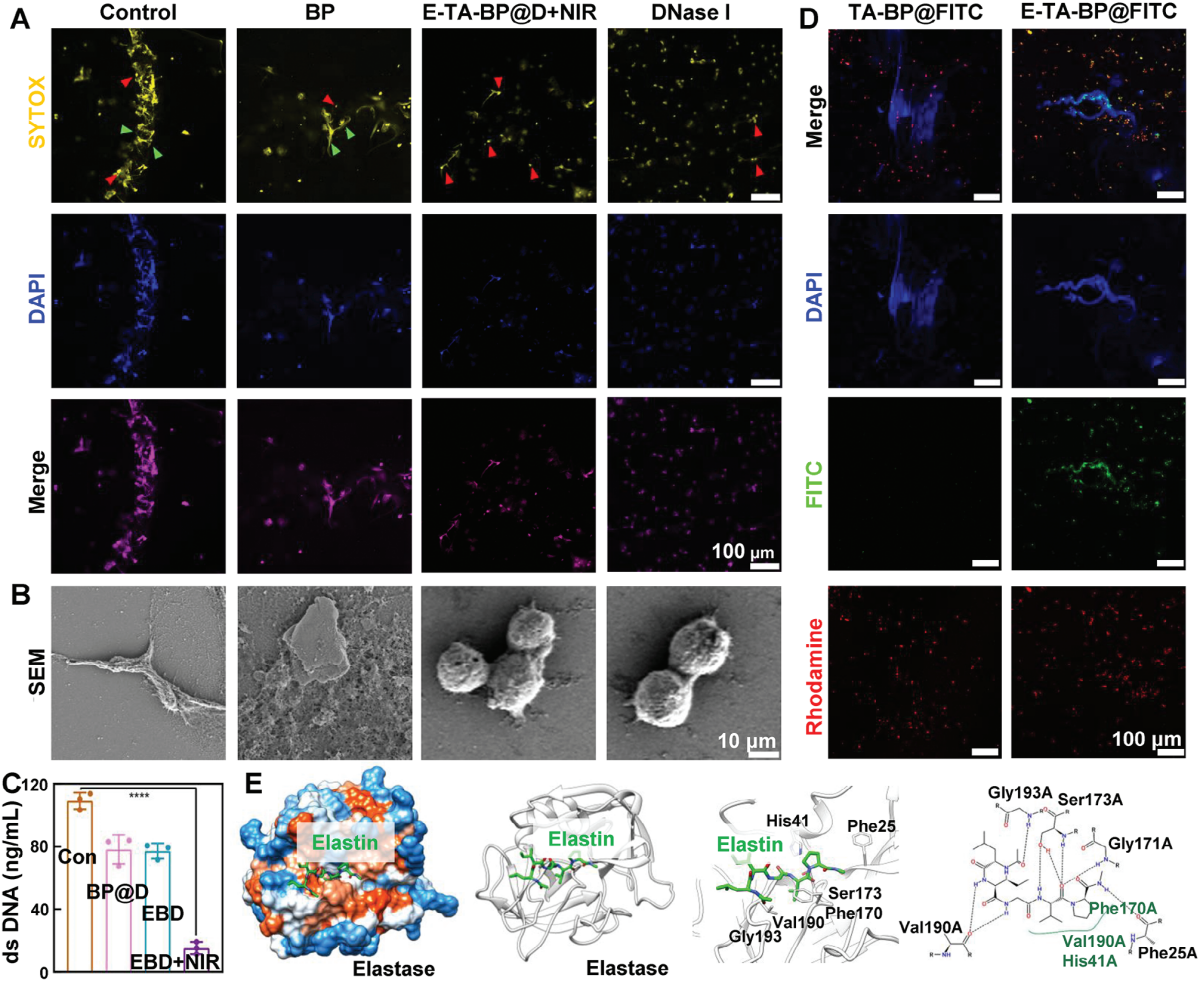
E‐TA‐BP@D functions as a targeted NET scavenger under NIR light illumination. A) Representative fluorescent microphotographs of NETs. B) Typical SEM images of NETs. C) Quantification of NETs in different treatment groups. Data are presented as mean ± SD (*n* = 3) and analyzed using a one‐way ANOVA, ****p < 0.0001. Con: Control, EBD: E‐TA‐BP@D, and EBD+NIR: E‐TA‐BP@D+NIR. D) Fluorescence images in TA‐BP@FITC, E‐TA‐BP@FITC groups where NETs were present. E) The predicted binding mode of elastin with human neutrophil elastase. Protein was illustrated as surface model (first from left) or ribbon model (second from left). The structure of elastin was shown as green sticks. Third from left: the detailed interactions between elastin and surrounding residues in human neutrophil elastase, where protein is shown as ribbon model, elastin is shown as green sticks, and interacting residues are shown as white sticks. Fourth from left: the 2D protein–ligand interactions.

The NETs’ digestion efficacy of E‐TA‐BP@D under NIR light illumination was evaluated using CLSM and SEM. In response to stimulation with phorbol 12‐myristate 13‐acetate (PMA), neutrophils extruded decondensed chromatin to form web‐like NETs. As shown in **Figure** **4**A, neutrophils stimulated with PMA produced abundant NETs (green arrows) with both blue and yellow fluorescence, indicating consistent localization. The cell impermeable fluorescent DNA dye SYTOX YELLOW also labeled neutrophil nuclei (red arrows), indicating neutrophil death accompanied by NET formation. Following DNase I treatment, the DNA mesh‐like structures were barely visible and the E‐TA‐BP@D+NIR group exhibited similar results. A significant number of NETs was observed in the BP group. The quantitative analysis of yellow fluorescence, as depicted in Figure S17, Supporting Information, reveals a notable trend. Both the E‐TA‐BP@D+NIR and DNase groups demonstrated a significant decrease in the quantity of NETs when compared to the control group. Similar results were observed in the SEM images. Only individual cells were visible in the DNase I and E‐TA‐BP@D+NIR groups, whereas mesh‐like DNA structures, resembling NETs, were observed in the Control and BP groups (Figure 4B). Subsequently, we quantitatively assessed the effect of E‐TA‐BP@D+NIR on the NETs using a DNA concentration assay kit.^[^ ^34^ ^]^ The DNA concentration in the E‐TA‐BP@D+NIR group was significantly decreased, with an 86 ± 3.5% reduction compared to that in the Control group (Figure 4C). These results indicated that DNase I released by E‐TA‐BP@D under NIR light irradiation remained active and effectively eliminated NETs.

The targeted ability of E‐TA‐BP@D toward NETs was evaluated by fluorescence colocalization. After co‐culturing E‐TA‐BP@Fluorescein isothiocyanate (FITC) or TA‐BP@FITC with NETs, good co‐localization was observed between elastase (red fluorescence) and E‐TA‐BP@FITC (green fluorescence), indicating that E‐TA‐BP@FITC effectively targeted elastase through elastin. Significant aggregation of E‐TA‐BP@FITC was also observed in elastase‐mediated NETs. In comparison, in the field of view where NETs were present, nearly no green fluorescence of TA‐BP@FITC was observed, and in the field of view where TA‐BP@FITC was present, no NETs were observed, indicating that TA‐BP@FITC did not effectively target NETs (Figure 4D; Figure S18, Supporting Information). The results in Figure 4A–D; Figure S18, Supporting Information indicated that E‐TA‐BP@D under NIR light irradiation could target elastase on NETs through the E component and realize the targeted degradation of NETs.

Computational simulation results for elastin‐targeted binding to elastase are depicted in Figure 4E. The top‐scoring result was selected to represent the favorable binding mode of the elastin peptide against human neutrophil elastase. The elastin peptide fitted well within the active site cavity of the elastase. The acetyl groups of the elastin peptide formed hydrogen bonds with Gly193 in elastase. The Ile and Gly groups from the elastin peptide formed hydrogen bonds with Val190 in elastase. The Val groups of the elastin peptide formed hydrogen bonds with Gly171 and Ser173 in elastase. The amino groups of the elastin peptide formed hydrogen bonds with Phe25 in elastase. In addition, hydrophobic interactions were observed between the elastin peptide and His41, Phe170, and Val190 in the elastase. These interactions contributed to the targeted binding of the elastin peptide with elastase. Table S1, Supporting Information displays the protein–ligand interaction scores calculated using different scoring functions, which represent the binding ability evaluated by theoretical calculations.

### 2.5 E‐TA‐BP@D With NIR Light Irradiation Rectifies the Biased Immunoreactivity Caused by Accumulated Nets

**Figure 5:**
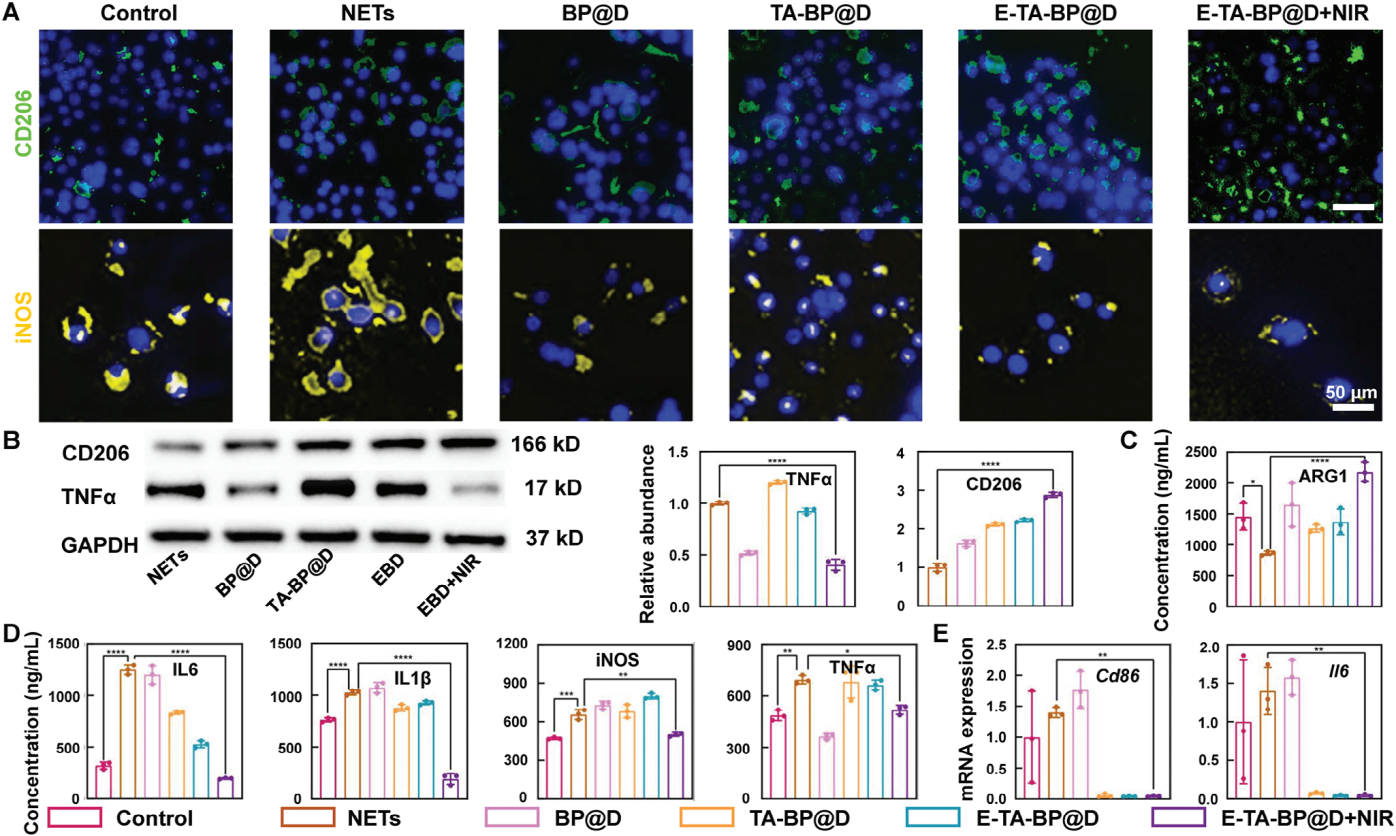
E‐TA‐BP@D with NIR light irradiation rectifies the biased immunoreactivity caused by accumulated NETs. A) Immunofluorescence staining images of CD206 and iNOS. B) Western blot images and quantification of western blot bands. C) ELISA results of ARG1. D) ELISA results of IL6, IL1β, iNOS, and TNFα. E) qPCR results of *Cd86* and *Il6*. Data are presented as mean ± SD (*n* = 3) and analyzed using a one‐way ANOVA, *p < 0.05, **p < 0.01, ***p < 0.001, and ****p < 0.0001.

Immunofluorescence staining, western blotting, enzyme‐linked immunosorbent assay (ELISA), and quantitative polymerase chain reaction (qPCR) are used to assess the alleviation of NETs‐induced inflammation. CD206 and arginase (ARG1) are closely related to the M2 (anti‐inflammatory) phase macrophage; while, tumor necrosis factor α (TNFα), interleukin (IL) 1β, CD86, IL6, and inducible nitric oxide synthase (iNOS) are closely related to the M1 (proinflammatory) phase macrophage.^[^ ^35^ ^]^ Immunofluorescence staining demonstrates a reduction in CD206 positive area and an increase in the iNOS positive area in the NETs group, whereas a significant increase in CD206 positive area and a noticeable reduction in the iNOS‐positive area are observed in the E‐TA‐BP@D+NIR group (**Figure** **5**A). Western blot results show that, after treatment with E‐TA‐BP@D+NIR, RAW 267.4 cells display a significant decrease in TNFα expression and an upregulation of CD206 expression compared with the NETs group (Figure 5B). ELISA demonstrates similar results. The expression of IL6, IL1β, iNOS, and TNFα is upregulated; while, the expression of the M2‐associated cytokine ARG1 is reduced in the NETs group, compared with in the Control group. After E‐TA‐BP@D+NIR treatment, the opposite changes are detected (Figure 5C,D). qPCR results are displayed in Figure 5E. Upon NETs stimulation, the transcriptional levels of *Cd86* and *Il6* are upregulated. However, treatment with E‐TA‐BP@D+NIR significantly reduces the translation of *Cd86* and *Il6*. These results indicate that NETs exacerbate inflammation, which can be effectively reversed by E‐TA‐BP@D+NIR treatment by promoting macrophage transition from the M1 phenotype to the M2 phenotype.

### 2.6 Both the Elemental Component and the Immunity Modulation Capacity of E‐TA‐BP@D Promote Osteogenic Differentiation of BMSCs

**Figure 6:**
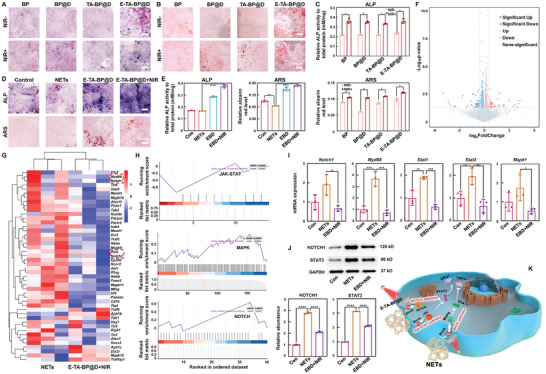
Osteogenic differentiation of BMSCs, RNA sequencing, and cellular validation. A) ALP assay images in different treatment groups. B) ARS staining of in vitro calcium deposits. C) Quantification of ALP or ARS staining in A&B. D) ALP and ARS images of different conditioned media groups. E) Quantification of ALP or ARS expression in (D). F) Volcano plot of differentially expressed genes (foldchange ≥ 1.5, *p* < 0.05). G) Cluster heatmap of differentially expressed genes of interest. H) Gene set enrichment analysis identified the JAK‐STAT, MAPK, and NOTCH signaling pathways as significantly varied pathways. I) qPCR results of *Notch1*, *Myd88*, *Stat1*, *Stat3*, and *Mapk1*. J) Western blot images of NOTCH1 and STAT3, along with the quantification of the western blot bands. K) Schematic diagram illustrating RNA sequencing and cellular validation results. Data are presented as mean ± SD (*n* = 3) and analyzed using a one‐way ANOVA, *p < 0.05, **p < 0.01, ***p < 0.001, and ****p < 0.0001. Con: Control, EBD: E‐TA‐BP@D, and EBD+NIR: E‐TA‐BP@D+NIR.

The osteogenic differentiation ability of the BP‐based nanocapsules was assessed using alkaline phosphatase (ALP) and Alizarin Red S (ARS) staining. The BP, BP@D, TA‐BP@D, and E‐TA‐BP@D groups without NIR light irradiation demonstrated certain levels of osteogenic differentiation. In addition, under NIR light irradiation, an appropriate temperature synergistically accelerated osteogenic activity (**Figure** **6**A–C), mainly owing to the dual pro‐biomineralization properties of BP. On the one hand, the photothermal effect of BP leads to an increase in temperature, which in turn, promotes biomineralization. On the other hand, the degradation products of BP, phosphate ions, provide a phosphorus source for biomineralization. Previous studies have reported that thermal energy activates the heat shock response in osteoblasts to enhance their migration and osteogenic differentiation capabilities.^[^ ^36^, ^37^ ^]^ NIR light can accelerate the decomposition of BP into phosphate ions, providing more phosphorus sources for biomineralization.^[^ ^38^ ^]^

After 14 d of osteogenic induction using conditioned medium, the E‐TA‐BP@D+NIR group exhibited the deepest staining and highest ALP activity, followed by the E‐TA‐BP@D and Control groups, whereas the NETs group displayed the least coloration. Similar results were observed for ARS staining (Figure 6D,E). These findings indicate that the polarization of macrophages induced by NETs was significantly altered by E‐TA‐BP@D+NIR, resulting in a favorable microenvironment for osteogenic differentiation and tissue formation (Figure S19, Supporting Information).

### 2.7 Several Signaling Pathways Play Vital Roles in Immune Regulation Process

According to the results analyzed by OmicStudio tools, a total of 83 differentially expressed genes (foldchange ≥ 1.5,^[^ ^39^, ^40^ ^]^ *p* < 0.05) were identified, with 37 upregulated and 46 downregulated, exhibiting highly significant expression patterns (Figure 6F). A heatmap of the selected genes of interest demonstrated that E‐TA‐BP@D+NIR could alter macrophage polarization from the M1 toward M2 status and inhibit inflammation‐related genes, such as *Myd88*, *Notch1*, *Rela*, and *Notch2*, associated with the JAK‐STAT, MAPK, and NOTCH signaling pathways (Figure 6G). The gene ontology (GO) enrichment analysis revealed that E‐TA‐BP@D+NIR modulated immune response and extracellular matrix (Figure S20, Supporting Information). The Kyoto Encyclopedia of Genes and Genomes (KEGG) analysis revealed a significant enrichment of the JAK‐STAT, MAPK, and NOTCH signaling pathways, underscoring their pivotal roles in the E‐TA‐BP@D+NIR group, particularly in facilitating inflammation resolution and cellular recovery (Figure S21, Supporting Information). In addition, gene set enrichment analysis identified these same three pathways as significantly altered in the E‐TA‐BP@D+NIR treatment group (Figure 6H). In the cellular experiments, the results of Western blot and qPCR validated the RNA sequencing findings. Compared to the Control group, the NETs group exhibited a significant increase in the transcription or expression of key genes or proteins involved in three signaling pathways. In contrast, the E‐TA‐BP@D+NIR group reversed this trend, reducing the levels to a level comparable to the Control group (Figure 6I,J). These findings are summarized in a schematic diagram (Figure 6K). Based on the results of computational simulation, RNA sequencing, and cellular validation, the mechanisms of how the nanocapsules interact with NETs are summarized into a schematic diagram in Figure S22, Supporting Information.

### 2.8 E‐TA‐BP@D+NIR Demonstrates Good Efficacy in Two Chronic Infectious Bone Destruction Disease Models

**Figure 7:**
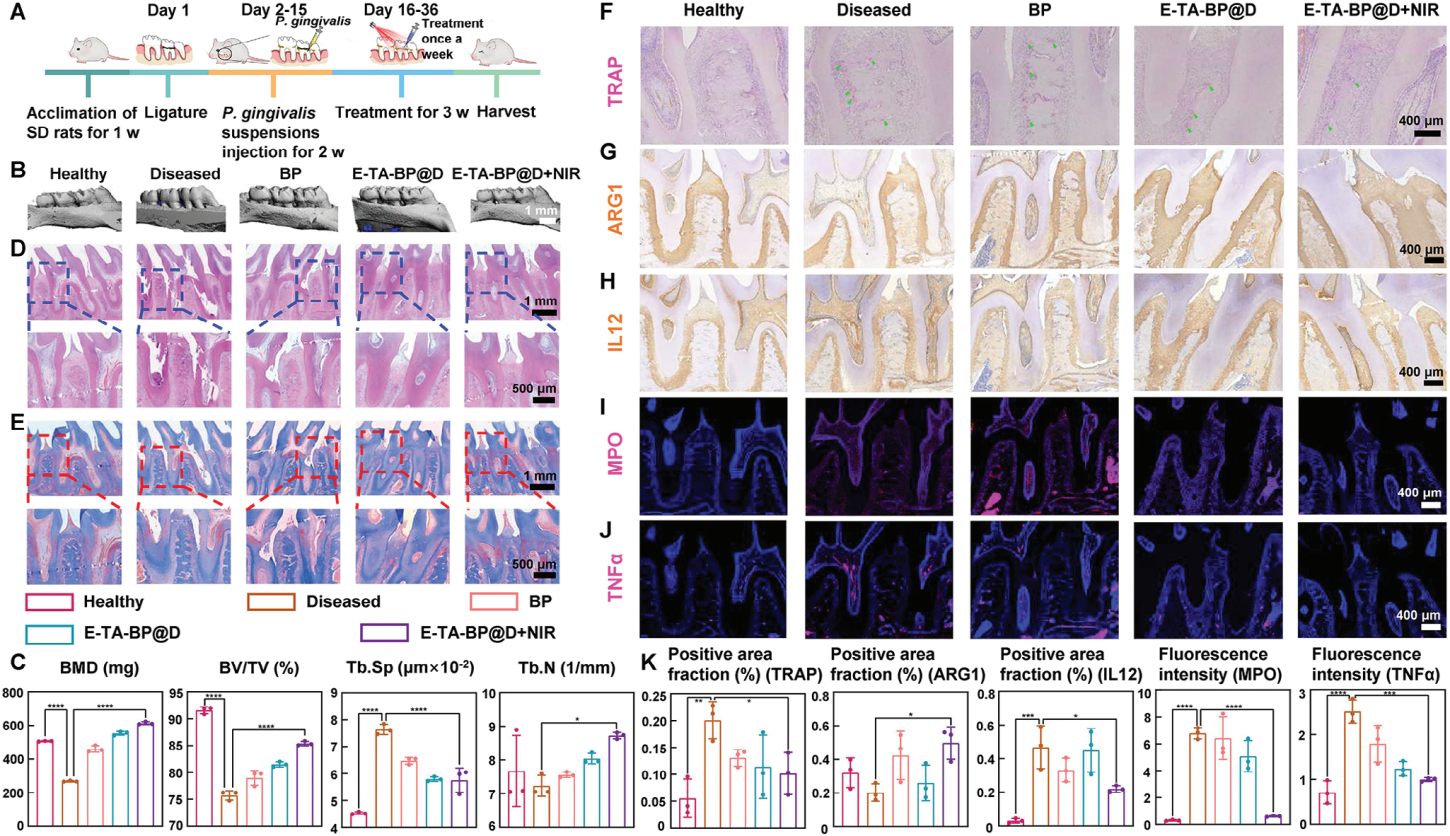
In vivo therapeutic efficiency for periodontitis. A) Schematic diagram of the animal experiments. B) The 3D reconstruction sections along the longitudinal direction of the maxillae in micro‐CT assay. C) Quantification of micro‐CT assay. D) H&E‐stained paraffin sections. E) Masson's trichrome staining. F–H) Immunohistochemical staining: TRAP, ARG1, and IL12. I,J) Immunofluorescence staining: MPO, TNFα. K) Quantitative analysis results of immunohistochemical and immunofluorescence staining. Data are presented as mean ± SD (*n* = 3) and analyzed using a one‐way ANOVA, *p < 0.05, **p < 0.01, ***p < 0.001, and ****p < 0.0001.

Surgical procedures used in the periodontitis model are shown in **Figure** **7**A. No oral mucosal lesions were detected (Figure S23, Supporting Information), and no pathological damage was observed in the major organs of the rats (Figure S24, Supporting Information), indicating good biocompatibility of E‐TA‐BP@D. After treatment, bone regeneration around the maxillary second molars was evaluated using micro‐computed tomography (micro‐CT). The diseased group exhibited a significantly lower bone volume, fewer trabeculae, lower trabecular thickness, and higher trabecular separation than the healthy group. Bone destruction was successfully repaired in all treatment groups, with the E‐TA‐BP@D+NIR group showing the most significant efficacy (Figure 7B,C). As mentioned in Section 2.6, the temperature increase caused by the photothermal effect promoted osteogenesis through two mechanisms: directly by the elevated temperature itself and indirectly by enhancing the degradation of phosphate groups, which in turn promoted osteogenesis.^[^ ^36^, ^37^, ^38^ ^]^ As depicted by hematoxylin and eosin (H&E) staining, the junctional epithelium was attached distally to the cemento–enamel junction with infiltration of inflammatory cells in the diseased group, whereas tissue abnormality was alleviated with E‐TA‐BP@D+NIR treatment (Figure 7D). In immunohistochemical staining, the E‐TA‐BP@D+NIR group showed lower levels of the neutrophil‐related marker, monocyte chemotactic protein 1 (MCP1), compared to the Diseased group (Figure S25, Supporting Information). In conjunction with the H&E staining results, it could be observed that the E‐TA‐BP@D+NIR group had fewer neutrophils than the Diseased group. Masson's staining revealed that the collagenous fibers were denser and more structured in the E‐TA‐BP@D+NIR group, similar to the normal tissues in the healthy group. Conversely, deteriorated fibers with inflammatory responses were observed in the diseased group (Figure 7E). Immunohistochemical staining for tartrate resistant acid phosphatase (TRAP), runt‐related transcription factor 2 (RUNX2), osteocalcin (OCN), osteopontin (OPN), ARG1, and IL12 was performed to further evaluate the osteoclastic or osteogenic activity and inflammatory status in the periodontal tissues of the different groups. The diseased group showed a higher level of TRAP‐positive markers than the healthy group, whereas the E‐TA‐BP@D+NIR group reversed this trend, indicating a compromised osteoclastic capability (Figure 7F). The diseased group showed fewer RUNX2, OPN, and OCN‐positive markers than the healthy group. However, the E‐TA‐BP@D+NIR group increased these markers, suggesting an osteogenic potential (Figure S26, Supporting Information). Similarly, E‐TA‐BP@D+NIR significantly reversed the changes in ARG1 and IL12 expression (Figure 7G,H). Immunofluorescence staining of myeloperoxidase (MPO) (the specific marker for neutrophils and NETosis^[^ ^14^, ^41^, ^42^ ^]^) in the periodontal tissues yielded similar results, as well as TNFα. The diseased group showed a higher positive expression of MPO and TNFα compared with the healthy group; while, the E‐TA‐BP@D+NIR group reversed the trend, indicating the degradation of NETs in inflammatory lesions and the promotion of inflammation resolution (Figure 7I,J). The quantitative analysis results of immunofluorescence and immunohistochemistry are depicted in Figure 7K.

The results of the periapical periodontitis model, another chronic infectious bone destruction disease model, resembled those of the periodontitis model. H&E staining indicated a good biocompatibility of E‐TA‐BP@D (Figures S23 and S24, Supporting Information). The surgical procedure is summarized in Figure S27, Supporting Information. The E‐TA‐BP@D+NIR group exhibited a significant bone repair effect (Figure S28, Supporting Information). As shown by H&E and Masson's staining, infiltration of inflammatory cells and degenerated fibers was detected in the periapical tissues of the diseased group, which was alleviated in the E‐TA‐BP@D+NIR group (Figures S29 and S30, Supporting Information). Immunohistochemical and immunofluorescence staining indicated ablation of NETs in inflammatory lesions and promotion of inflammation resolution and bone remodeling (Figures S31–S34, Supporting Information).

## 3 Discussion

Following the initial enthusiasm for the discovery of the antimicrobial ability of neutrophils, the ability of NETs to kill microbes was questioned. In contrast, NETs are reportedly the dark side of neutrophils that directly kill epithelial and endothelial cells.^[^ ^7^, ^8^ ^]^ Although neutrophils may not exist at chronic inflammation sites, their remnants may magnify the inflammatory response beyond their fleeting lifetime in tissues.^[^ ^43^ ^]^ Studies to date have not fully paid attention to NETs regarding the design of biomaterials against chronic infections. We innovatively targeted NETs to combat chronic infectious bone destruction diseases in the present study.

In summary, our results reveal that the dual‐responsive DNase I‐releasing nanocapsule, E‐TA‐BP@D, was successfully prepared and has good biocompatibility and antibacterial efficiency through photodynamic as well as photothermal performance. E‐TA‐BP@D achieved targeted degradation of NETs, further rectifying the biased immune response. The immunomodulatory effect and BP's inherent pro‐osteogenic effect simultaneously advanced BMSCs osteogenic differentiation and repaired bone destruction in two animal models. The issue concerning defective bone repair in chronic infections, which was caused by biased host response brought about by overactivated NETs, was successfully resolved.

The bone tissue undergoes continuous building and degradation. Inflammation caused by chronic infections interferes with bone metabolism, promotes bone destruction, and impairs bone formation. Bacterial infections, immune disorders, and bone loss can persist in inflammatory niches. These three pathological states are dynamically intertwined and interact with each other, which is the root cause of difficulties in treatment.^[^ ^3^, ^13^, ^44^ ^]^ Previous studies have typically approached the control of bacterial infection or immune regulation in a one‐sided manner to treat chronic infectious bone destruction.^[^ ^45^, ^46^ ^]^ We formulated E‐TA‐BP@D to eliminate pathogens and modulate immune disarray caused by NETs, along with promoting bone defect repair. Chronic infectious bone destruction diseases include periodontitis, periapical periodontitis (two animal models used in the present study), open alveolar bone defect, chronic osteomyelitis, and chronic epiphysitis. The correlation between periodontitis and systemic diseases has been an active research field for several decades.^[^ ^11^ ^]^ Studies have demonstrated that NETs appear to link periodontitis with systemic diseases as peptidyl arginine deiminases released from neutrophils during the production of NETs result in a sustained, self‐sustaining local immune reaction. The primary immunogenic role of NETs is associated with the activity of peptidyl arginine deiminase 4, which catalyzes the citrullination of histones and other molecules such as α‐enolase, potentially leading to a breakdown of immune tolerance.^[^ ^13^ ^]^ Therefore, we chose periodontitis as a major animal model to test the material efficacy fighting chronic infectious bone destruction diseases.

DNase I, which cleaves DNA via an endonucleolytic approach, has emerged as a prospect for disrupting NETs to block pathological damage in a chronic inflammatory microenvironment.^[^ ^7^, ^47^ ^]^ Nevertheless, it is not suitable to administer DNase I systemically because of its short half‐life,^[^ ^48^, ^49^ ^]^ and the systematic inhibition of NETs possibly leads to enhanced vulnerability to bacterial infection because it impacts the inherent defense of the immune system.^[^ ^50^ ^]^ Therefore, it is highly desirable to realize locally targeted delivery and responsive release of DNase I to disrupt NETs within an inflammatory niche. Previous studies have designed nanoparticles for the local delivery of DNase.^[^ ^17^, ^51^ ^]^ In our present study, dual‐responsive DNase I release to NIR light and NETs realizes the double need of active therapy and intelligent trigger, respectively. Molecular simulations have been conducted to gain fundamental understanding on the unique targeting process. Hydrogen bonds and hydrophobic interactions between amino acids mainly lead to the targeting effect. TA coating, as a mussel‐inspired biomimetic, not only isolates BP from oxygen and water to improve its stability but also aids in further coupling E to target NETs.^[^ ^23^ ^]^ The E and TA modifications simultaneously enhanced the biocompatibility of BP.^[^ ^23^ ^]^ NETs induce a proinflammatory M1 macrophage phenotype, which is consistent with previous findings.^[^ ^52^ ^]^ However, under NIR light irradiation, E‐TA‐BP@D reversed the proinflammatory effect of NETs on macrophages, inducing a phenotypic shift from M1 to M2. The immunomodulatory microenvironment associated with M2 phenotype facilitates tissue generation and repair. In line with our RNA‐seq findings, key genes/proteins involved in the JAK‐STAT, MAPK, and NOTCH pathways, such as *Notch1*, *Mapk1*, and *Stat3*, were downregulated following treatment with E‐TA‐BP@D+NIR.^[^ ^53^, ^54^, ^55^ ^]^ Moreover, owing to its inherent capacity for spontaneous oxidation and phosphate production, BP serves as a source of phosphorus to support osteogenic activity.^[^ ^56^, ^57^ ^]^ Fine‐tuning of tissue‐specific immune responses plays a major role in maintaining tissue homeostasis.^[^ ^15^ ^]^ The effects of both immunomodulatory microenvironmental changes and BP promote the osteogenic differentiation of BMSCs, and in turn, enhance local bone healing.

Although cell death is a routine process, it can be pathological and exacerbate chronic inflammation and disease. Therefore, targeting cell death may have the potential to combat chronic inflammation.^[^ ^58^ ^]^ This study contributes novel theories and ideas for regulating biased immunological disturbances in chronic infectious diseases and treating chronic infectious bone destruction. E‐TA‐BP@D may be used for the local treatment of various chronic infectious bone destruction diseases, such as in periodontitis as a periodontal irrigation solution, in periapical periodontitis as an endodontic irrigation solution or as a root canal filling paste. The present study still has limitations. In addition to DNA and neutrophil elastase, NETs contain other protein components, and it is worth further research to determine whether these other components have biological effects and whether the nanocapsules interact with biological pathways that were not explored in this study. Furthermore, as a double‐edged sword, NETs may play different roles at different stages of disease progression. For example, a study has suggested that aggregated NETs may limit acute inflammation by degrading cytokines and chemokines.^[^ ^59^ ^]^ Therefore, the long‐term efficacy of the nanocapsules is more complex and still warrants further research.

## 4 Conclusion

A dual‐responsive nanocapsuled system, E‐TA‐BP@D, was synthesized. E‐TA‐BP@D accomplished antibacterial efficacy within infection niche and achieved targeted degradation of NETs. The immunocorrective effect and its innate pro‐osteogenic property together promoted bone repair. This study sheds important new light on rectifying biased immunological dysfunction when combating chronic infections.

## 5 Experimental Section

### Synthesis and Characterization of E‐TA‐BP@D

A liquid exfoliation method was used to prepare the BP nanosheets,^[^ ^24^ ^]^ and the detailed procedures are described in the Supporting Information. One mg BP nanosheets was dispersed in 0.2 mL ethanol, to which a solution of hydrochloric acid (4 m, 20 µL) and DNase I (5 mg mL^−1^) (Solarbio, China) was added. The mixture was continuously oscillated in an incubator (120 rpm) at 37 °C for 2 h. The precipitate (BP@D) was collected by centrifugation at 9000 rpm for 5 min. The DNase I load efficiency was calculated using a previously published method.^[^ ^60^ ^]^ TA was encapsulated through a complexation reaction to obtain TA‐BP@D,^[^ ^61^ ^]^ and E was modified via a non‐covalent bonding reaction to obtain E‐TA‐BP@D;^[^ ^62^ ^]^ the detailed procedures are described in the Supporting Information.

The morphology of the BP‐based nanocapsules was examined using TEM (Tecnai G2 F30, FEI, USA), FE‐SEM (GeminiSEM 300, ZEISS, Germany), and AFM (Dimension Icon, Bruker, USA). Chemical structure was characterized using XPS (Axis HSi, Kratos, UK) with Al Kα radiation (1486.6 eV photons, 150 W) as the X‐ray source for excitation. Size and zeta potential were measured using a laser particle size instrument (Zetasizer Nano ZS90, Malvern, UK). The sizes of BP and E‐TA‐BP@D in water and air were consecutively measured for 7 d as a stability test.^[^ ^26^ ^]^ The release of DNase I in response to different stimuli was measured using the absorbance method.^[^ ^61^ ^]^ The light absorption capacity of the BP‐based nanocapsules near NIR wavelengths was detected using UV–vis DRS (Cary 7000, Agilent, USA) with BaSO_4_ as the background.

### Photothermal and Photodynamic Performance Measurement

The photothermal performance of the BP‐based nanocapsules was evaluated in phosphate‐buffered saline (PBS) at room temperature. A thermal infrared camera (E6, FLIR, Sweden) was used to capture infrared thermal images and measure the real‐time temperature. BP‐based nanocapsules were illuminated with 808 nm NIR light at different densities (0.5, 1, 1.5 W cm^−2^). The variation in the temperature was recorded every 1 min interval. Furthermore, the stability of the photothermal property was evaluated by irradiating with a 1.5 W cm^−2^ NIR laser for three cycles of heating–cooling tests: 10 min for heating and 15 min for cooling.

To evaluate photodynamic efficiency, GSH depletion was measured using a microplate reader (Molecular Devices, USA). ROS generation was detected by spectrophotometry. DPBF (Aladdin, China) was employed to detect the generation of ^1^O_2_ and ·O_2_^−^. MB was utilized to detect the generation ·OH in a similar way to the detection of ^1^O_2_ and ·O_2_^−^. ESR (JES‐FA200, JEOL, Japan) was used to assess the type of ROS generated. After the BP‐based nanocapsules were irradiated with an 808 nm NIR laser for setting times (0, 5, and 10 min), different types of ROS were detected with different types of capture reagents. Further, 2,2,6,6‐tetramethylpiperi‐ dine‐1‐oxyl (Aladdin, China) was used to capture ^1^O_2_. Detailed GSH and DPBF depletion procedures are described in the Supporting Information.

### In Vitro Cytotoxicity and Antibacterial Efficiency Evaluation

HPLFs, hBMSCs, HOKs, L929 cells, and mBMSCs were used to evaluate the cytotoxicity. All procedures involving human tissue samples were approved and authorized by the Research Ethics Committee of West China Hospital of Stomatology (No. WCHSIRB‐D‐2021‐084). The proliferative activity of cells was evaluated using CCK‐8 (APE×BIO, USA). FITC‐phalloidin/DAPI staining was performed to evaluate the cytoskeletal structure of the cells. The morphology of the cells co‐cultured with the BP‐based nanocapsules was assessed using CLSM (N‐SIM, Nikon, Japan) and SEM (JEM1011, JEOL, Japan). Detailed procedures are described in the Supporting Information.

*P. gingivalis* and *E. faecalis* were used to assess the antibacterial efficiency of the BP‐based nanocapsules. The antibacterial efficiency was evaluated using plate counting and live/dead staining methods. The morphologies of the bacteria in the different groups were evaluated using SEM (JEM1011, JEOL, Japan) and TEM (Tecnai G2 F30, FEI, USA). Detailed procedures are described in the Supporting Information.

### Targeted Scavenging of NETs

The bone marrow extracted from 4–6‐week‐old KM mice was collected in Hanks balanced salt solution without Ca^2+^/Mg^2+^ (Solarbio, China). Neutrophils were isolated from the bone marrow using a mouse Neutrophil Isolation Kit (Solarbio, China) according to the manufacturer's protocol. Neutrophils isolated from the bone marrow were cultured in RPMI 1640 medium supplemented with 10% fetal bovine serum.^[^ ^63^ ^]^

Neutrophils (8 × 10^4^ cells mL^−1^, 1 mL) were placed on cell slides pretreated with poly‐L‐lysine (ProCell, China) to stimulate NETs fabrication. Next, 200 nm PMA (MedChemExpress, USA) was added for 4 h to induce NETs generation.^[^ ^4^ ^]^ Subsequently, PBS as a negative control, DNase I (50 U mL^−1^) (Solarbio, China) as a positive control, or other BP‐based nanocapsules in experimental groups were added to the culture medium for 1 h. The cell slides were stained with the cell‐impermeable fluorescent DNA dye SYTOX Yellow (Thermo Fisher Scientific, USA) or the cell‐permeable fluorescent DNA dye DAPI (Solarbio, China).^[^ ^9^ ^]^ NETs were observed with CLSM (N‐SIM, Nikon, Japan) and SEM (JEM1011, JEOL, Japan), and NETs were quantified using a Quant‐iT PicoGreen dsDNA Assay Kit (Thermo Fisher, USA).^[^ ^34^ ^]^ FITC (Thermo Fisher, USA) was used to label TA‐BP or E‐TA‐BP, where the mixture was oscillated in a constant temperature incubator (120 rpm) at 37 °C for 2 h, protected from light. PMA (200 nm) (MedChemExpress, USA) was added to plates with cell slides bedded on neutrophils (8 × 10^4^ cells mL^−1^, 1 mL) to induce NETs generation for 4 h. 100 µL TA‐BP@FITC or E‐TA‐BP@FITC was then added and incubated at 37 °C for 2 h in dark. The supernatant was discarded, and the cell slides were stained with DAPI (Solarbio, China) and elastase substrate (Z‐Ala‐Ala‐Ala‐Ala)2Rhodamine110, respectively.^[^ ^60^ ^]^ The cell slides were observed using CLSM (N‐SIM, Nikon, Japan).

To analyze the mechanism of elastin at the periphery of E‐TA‐BP@D targeted binding with elastase on NETs, a computational simulation analysis was performed.^[^ ^64^, ^65^ ^]^ Detailed procedures for the computational simulation analysis are described in the Supporting Information.

### In Vitro Assessment of Immunity Modulation Capacity

A mouse macrophage cell line (RAW 246.7) was seeded in 24‐well microtiter plates and treated with lipopolysaccharide (LPS) from *Escherichia coli* (Solarbio, China) for 6 h (Control group).^[^ ^66^, ^67^ ^]^ Subsequently, the cells were washed several times, and NETs were added and co‐incubated for 24 h (NETs group). The cells were then subjected to different treatments (experimental groups) twice, once for 1 d (NIR group: rested for 1 d after irradiation for 10 min), and finally, used to evaluate the immunity modulation capacity. qPCR was performed to detect the expression of immune‐related genes. The primer sequences used for qPCR are listed in Table S2, Supporting Information. Immunofluorescence staining was performed using primary antibodies against iNOS (M1 marker) and CD206 (M2 marker). Antibodies for TNFα, CD206 or β‐actin (1:2000, Thermo Fisher, USA) were used for western blot (Table S3, Supporting Information). The levels of TNFα, iNOS, IL1β, and ARG1 were evaluated using Quantikine ELISA kits (R&D, USA).^[^ ^35^ ^]^ All kits were used according to the manufacturers’ instructions.

### Osteogenic Differentiation of BMSCs Induced by BP‐Based Nanocapsules or Conditioned Medium

RAW 246.7 was seeded in 24‐well microtiter plates and treated with LPS for 6 h. Subsequently, NETs were added and the cells were co‐incubated for 24 h. The cells were then subjected to different treatments for 3 d (NIR group: incubated for 3 d after irradiation for 10 min). The supernatant was collected and mixed with α‐MEM medium (1:3) prepared using sodium citrate (50 ng mL^−1^) (Solarbio, China), sodium β‐glycerophosphate (10 mm) (Solarbio, China), and dexamethasone (10 nm) (Solarbio, China), namely the conditioned medium.

BMSCs were seeded in 24‐well microtiter plates and cultured in different conditioned media or BP‐based nanocapsules for 14 d (NIR group: incubated for 14 d after irradiation for 10 min). ALP expression was evaluated using a BCIP‐NBT Kit (Beyotime, China) according to the manufacturer's instructions. Extracellular matrix mineralization was measured after ARS staining using a microplate reader (Molecular Devices, USA) at 562 nm.

### RNA Sequencing and Cellular Validation

Macrophages from the NETs and E‐TA‐BP@D+NIR groups in the “ In Vitro Assessment of Immunity Modulation Capacity” were subjected to RNA sequencing, and those from the Control, NETs, and E‐TA‐BP@D+NIR groups were used for cellular validation. Total RNA from RAW 264.7 was extracted using the TRIzol reagent (Thermo Fisher Scientific, USA). The RNA libraries were sequenced on an Illumina Nova–Seq 6000 (Illumina, USA) platform. Bioinformatics analysis was performed using the OmicStudio tools (https://www.omicstudio.cn/tool). To detect the expression of genes related to pathways identified by RNA sequencing, qPCR was performed. The primer sequences used for qPCR are listed in Table S2 , Supporting Information. Antibodies for NOTCH1, STAT3, or β‐actin (1:2000, Thermo Fisher, USA) were used for western blot (Table S3, Supporting Information).

### In Vivo Therapeutic Efficiency

All procedures concerning animals were approved and authorized by the Research Ethics Committee of West China Hospital of Stomatology (No. WCHSIRB‐D‐2022‐068). All surgical procedures followed the standard guidelines.

Six–eight‐week‐old male Sprague–Dawley (SD) rats (200–250 g) were used (Dossy Animal Center, China). 5‐0 silk line was slid into the proximal‐distal space of the right maxillary second molar and pressed into the gingival sulcus. The ligation wire was checked, and *P. gingivalis* suspension was injected in situ into the second maxilla of the rats every 2 d.^[^ ^68^, ^69^, ^70^ ^]^ After 2‐week modeling, the rats were used for further experiments.

In addition to periodontitis, another chronic infectious bone destruction disease model, periapical periodontitis, was also conducted. SD rats were placed in the supine position, a 1/4 ball drill was used to open the first right mandibular molar, and the root canal was observed directly under a microscope. The root canal was then unchoked using the #8 K file. The medullary cavity was exposed for 2 weeks. After 2‐week modeling, the rats were used for follow‐up experiments.^[^ ^71^ ^]^

Rats were randomly divided into four groups with different treatments: Control, BP, E‐TA‐BP@D, and E‐TA‐BP@D+NIR (*n* = 6). BP‐based nanocapsules were injected into the lesion area and irradiated with an 808 nm laser (1.5 W cm^−2^) for 20 min in the NIR group. The treatment was performed once a week. After 3 weeks, the rats were euthanized. The mandibles and maxillae were dissected and fixed in 4% paraformaldehyde for 48 h. The samples were examined using micro‐CT (µCT50, SCANCO, Switzerland) at voltage of 102 kV and a current of 100 mA. Sampling sections of the buccal and lingual mucosa and major organs (heart, liver, spleen, lung, and kidney) were stained with H&E for histological analysis to explore biocompatibility.

After micro‐CT analysis, the samples were sequentially dehydrated with a graded ethanol series (70–100%), and then, encapsulated in a methyl methacrylate solution at 37 °C for 1 week. Sections (5 µm in thickness) were then prepared along the vertical direction of lesion using a microtome (Leica, Germany), and H&E and Masson's staining was performed. Immunohistochemical staining for TRAP, ARG1, IL12, MCP1, RUNX2, OCN, and OPN was performed to further demonstrate the inflammatory status and osteoclast or osteogenic activity in the lesion area. Immunofluorescence staining for TNFα and MPO (the specific marker for neutrophils and NETosis^[^ ^14^, ^41^, ^42^ ^]^) was performed. The antibodies used are listed in Table S3, Supporting Information.

### Statistical Analysis

Data are presented as the means ± SDs. Statistical analyses were conducted using SPSS software (version 21.0, IBM, USA). Statistical significance was evaluated using the unpaired Student's *t*‐test when two groups were compared at a value of *p* = 0.05. One‐way analysis of variance was conducted to detect significant effects of the variables when the number of groups was equal to or greater than three. Student–Newman–Keuls multiple comparison tests were performed at *p* = 0.05. For each representative experiment, at least three repetitions were measured.

## Conflict of Interest

The authors declare no conflict of interest.

## Author Contributions

S.T. and Y.Y. contributed equally to this work and are the co‐first authors. S.T. contributed to conceptualization, investigation, methodology, and writing‐original draft. Y.Y. contributed to investigation, methodology, and writing‐original draft. C.W. and J.Y. contributed to investigation and methodology. Z.W. and F.Z. contributed to methodology. K.L., Y.D., and Jia. L. contributed to Investigation. Jiy.L. contributed to conceptualization, funding acquisition, supervision, and writing‐review and editing.

## Supporting information



Supporting Information

## Data Availability

The data that support the findings of this study are available from the corresponding author upon reasonable request.
