# Supplementary material for: Nanocapsuled Neutrophil Extracellular Trap Scavenger Combating Chronic Infectious Bone Destruction Diseases
Source: Adv Sci (Weinh). 2025 Jan 17;12(10):2411274. doi: 10.1002/advs.202411274 (PMC11904938; doi:10.1002/advs.202411274)
Supplement: Supplementary file 1 — Supporting Information [file ADVS-12-2411274-s001.docx]

Supporting Information

Nanocapsuled neutrophil extracellular trap scavenger combating chronic infectious bone destruction diseases

Siying Tao^a, 1^, Yingming Yang ^a, 1^, Chenzhou Wu^b^, Jiaojiao Yang^c^, Ziyou Wang^a^, Fangjie Zhou^a^, Kunneng Liang^a^, Yi Deng^d^, Jianshu Li^e^, Jiyao Li^a, *^

**Experimental Section**

**Synthesis and characterization of E-TA-BP@D**

***Preparation of black phosphorus (BP) nanosheets***

In brief, bulk BP (15 mg) (11 tech, China) was added to the saturated NaOH/NMP solution. The saturated NaOH/NMP solution was used to confine the oxidation of BP. The mixture solution was then sonicated in ice bath for 9 h with an ultrasonic cleaner. Subsequently, the suspension was centrifuged at 2000 rpm for 15 min to move the remanent bulk BP particles. The supernatant was collected and centrifuged at 12000 rpm for 5 min to obtain BP nanosheets. The excrescent bulk BP particles was stored under 4℃ for further use.

***Tannic acid (TA) encapsulation***

1 mg of BP@D was suspended in 1 mL ethanol. Then 100 μL TA (40 mg/mL, pH>8) (Aladdin, USA) was added. The mixture was tempestuously shaken for 10 s. 100 μL Fe(NO_3_)_3_ (6 mg/mL) was subsequently added. Afterwards, the precipitate was collected by centrifugation at 12000 rpm for 5 min and lyophilized to obtain purified TA-BP@D.

***Elastin (E) encapsulation***

The prepared TA-BP@D (1 mg) was added with the solution of elastin (10 mg/mL, in 10.0 mM Tris-HCl buffer solution, pH 8.4) (Ryon Biological Technology, China). The mixture solution was then oscillated in the constant temperature incubator (120 rpm) at 37℃ for 1 h. Ultimately, the precipitate was collected by centrifugation at 12000 rpm for 5 min and lyophilized again to obtain E-TA-BP@D powders.

**Photodynamic performance measurement**

Glutathione (GSH) solution (400 μL, 10 mg/mL) (Sigma, USA) and BP-based nanocapusules were added to a well plate. After incubation at room temperature for 15 min, the mixtures were illuminated with a near-infrared (NIR) light for 10 min. Mixtures containing H_2_O_2_ (1 mM, Aladdin, USA) solution was utilized as positive control. The depletion of GSH was reflected by the decrease of absorbance at 410 nm, with adding Tris-HCl (5 mM, 450 μL) and 5,5'-dithiobis-(2-nitrobenzoic acid) solution (10 mM, 120 μL).

Different BP-based nanocapusules and 1,3-diphenylisobenzofuran (DPBF) (15 μM, 400 μL) solution were added in a well plate, and irradiated by NIR laser (808 nm, 1.5 W/cm^2^) for 10 min. The absorption of DPBF solution was measured by UV-Vis spectrophotometer (UV1800PC, AOE, China) at different setting times (0, 5, 10 min) with the spectrophotometer ranging from 300 nm to 500 nm.

***In vitro* cytotoxicity and antibacterial efficiency evaluation**

Mouse fibroblast (L929) or human oral keratinocyte (HOK) cell line was acquired from American Type Culture Collection (ATCC), and mouse bone marrow mesenchymal stem cells (mBMSCs) were extracted from the tibia of mice. Human periodontal ligament fibroblasts (hPLFs) were extracted from human periodontal ligaments derived from extracted tooth samples, while human bone marrow mesenchymal stem cells (hBMSCs) were obtained from human bone marrow derived from jaw bone fracture fragments. Five types of cells were cultured using Dulbecco’s modified Eagle’s media (DMEM) (Servicebio, China) and Minimum Essential Medium α (α-MEM) (Servicebio, China) supplemented with 10% fetal bovine serum (Gibco, USA) and 1% penicillin/streptomycin (Solarbio, China) at 37°C and 5% CO_2_. The initial density of cells inoculated with different BP-based nanosheets was 1×10^4^ cells/mL.

Cells were cultivated with different BP-based nanocapusules for 5 d. The proliferation activity of cells was evaluated with Cell Counting Kit-8 (CCK-8) (APE×BIO, USA). Cell culture medium containing 10% CCK-8 reagent was co-incubated in darkness for 2 h on the 1st, 3rd, and 5th day. The OD value of reacted medium was measured with the microplate reader (Molecular Devices, USA) at 450 nm.

The morphology of cells co-cultured with BP-based nanocapusules was assessed. Different samples and 48-well cell slides were put in 48-well culture plates inoculated with cells at 37°C for 3 d. Cell slides were rinsed twice by phosphate-buffered saline (PBS) and immobilized with 4% formaldehyde tissue fixation solution for 1 h. Subsequently, Triton X-100 (0.1% v/v) was applied for 20 min, then FITC-phalloidin (Solarbio, China) and DAPI (Solarbio, China) were utilized to counterstain cytoskeleton and nucleus of cells. Finally, fluorescent images were recorded with confocal laser scanning microscope (CLSM) (N-SIM, Nikon, Japan). The morphology of cells was further observed using scanning electron microscopy (SEM). Cells were treated with the same methods above for 3 d. Subsequently, Cell slides were immobilized and continuously dehydrated with different ethanol solutions (30, 50, 70, 80, 90, 100%). The typical images of cells were captured by SEM (JEM1011, JEOL, Japan).

*Porphyromonas gingivalis* and *Enterococcus faecalis* were employed to assess the antibacterial efficiency of BP-based nanocapusules. Brain-heart infusion broth was utilized to culture bacteria.

The antibacterial efficiency evaluation was conducted with the plate counting method and live/dead staining method. Briefly, bacterial suspension (1×10^4^ CFU/mL, 200 mL) was co-incubated with different BP-based nanosheets in a well plate and treated with different treatments, where the NIR laser illumination groups were treated with 808 nm NIR light irradiation under a power density of 1.5 W/cm^2^ for 10 min, while the non-illumination groups were placed in darkness for 10 min. After that, 50 μL treated bacterial suspension was spread in LB agar evenly and incubated at 37℃ for 24 h. Typical images of the agar plates were captured with a digital camera. Antibacterial rate of the different groups was calculated using the equation below.

Antibacterial rate= (Ab-Ac)/Ab×100%, where AC represents the mean CFUs of the control group (PBS without NIR laser illumination) and Ab represents the mean CFUs of the experimental group.

Live&Dead Bacterial Staining Kit was further employed to measure the antibacterial efficiency. Bacterial suspension (1×10^6^ CFU/mL, 1 mL) and a 48-well cell slide were incubated for 12 h in a 48-well plate at 37℃. Next, the samples were treated in a similar way to the plate counting approach. The live/dead BacLight bacteria viability Kit (Beibokit, China) was utilized to dye the cell slides for 20 min, and then the samples were gently washed twice with deionized water for 5 min each time. Finally, the typical images of different samples were captured with CLSM (N-SIM, Nikon, Japan).

To investigate the morphologies of bacteria in different groups, the bacteria morphologies were evaluated with SEM (JEM1011, JEOL, Japan) and transmission electron microscopy (TEM) (Tecnai G2 F30, FEI, USA). The bacterial suspension (200 μL, 1×10^7^ CFU/mL) and cell slides mixed with BP-based nanosheets in a 48-well plate was irradiated/unirradiated by NIR laser (1.5 W/cm^2^) for 10 min. The treated cell slides were then collected and 2.5 % (v/v) glutaraldehyde was utilized to fix bacteria, and the cell slides were dehydrated with different ethanol solution (30, 50, 70, 80, 90, 100%). Finally, the samples were coated with gold and were observed by SEM. Meanwhile, the surplus bacterial suspension was collected and centrifuged at 12000 rpm for 10 min and then the supernatant was discarded. 2.5 % (v/v) glutaraldehyde was utilized to fix bacteria. TEM images of samples were obtained after staining with uranyl acetate and lead citrate.

**Targeted scavenging of NETs**

To analyze the mechanisms on elastin at the periphery of E-TA-BP@D targetedly binding with elastase on NETs. Computational simulation analysis was performed. The structure of elastin peptide was obtained from PubChem (CAS:9007-58-3). Three-dimensional structure of elastin peptide was generated using Ligand preparation module of Sybyl and optimized using Powell method with Gasteiger-Hückel charges. Protein structure was downloaded from RCSB database (PDB: 3Q76). Protein structure was prepared by adding hydrogens and charges. Protomol was generated based on active site residues, which was then subjected for Surflex-Docking. The generated docking pose was rescored using CScore method to evaluate the binding affinity of ligand with target protein. Figures were created using Pymol and Proteins Plus web server.

**Figures**


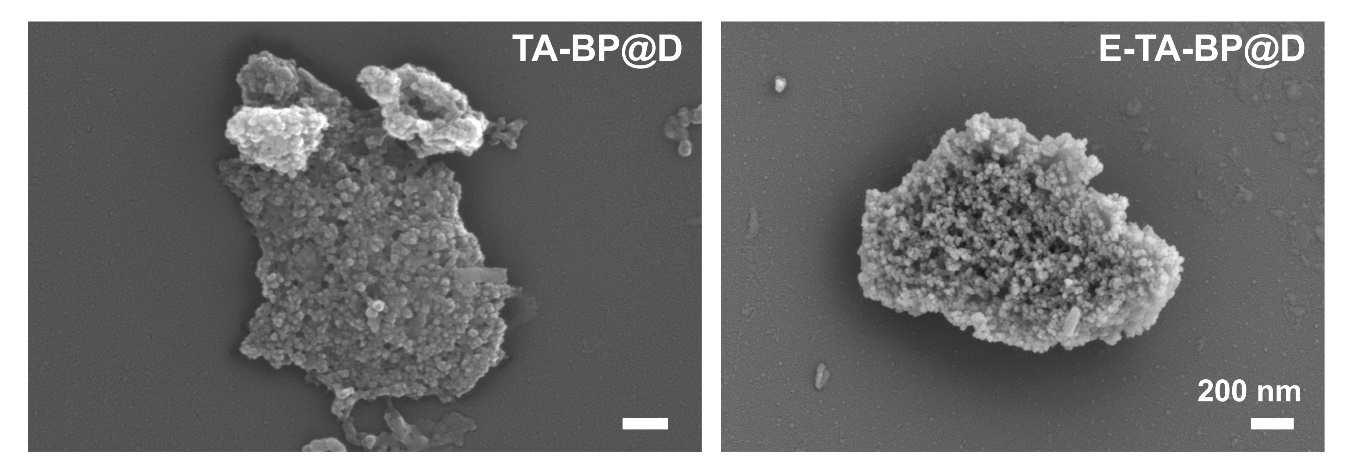


Figure S1. Characterization. Field emission scanning electron microscopy (FE-SEM) images of TA-BP@D and E-TA-BP@D.


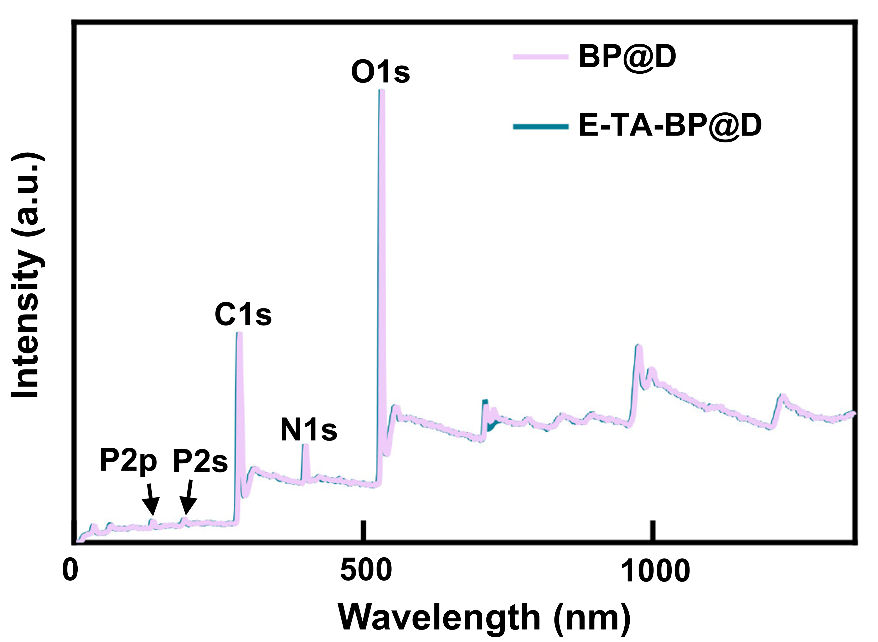


Figure S2. Characterization. X-ray photoelectron spectroscopy (XPS) spectra of BP@D and E-TA-BP@D.


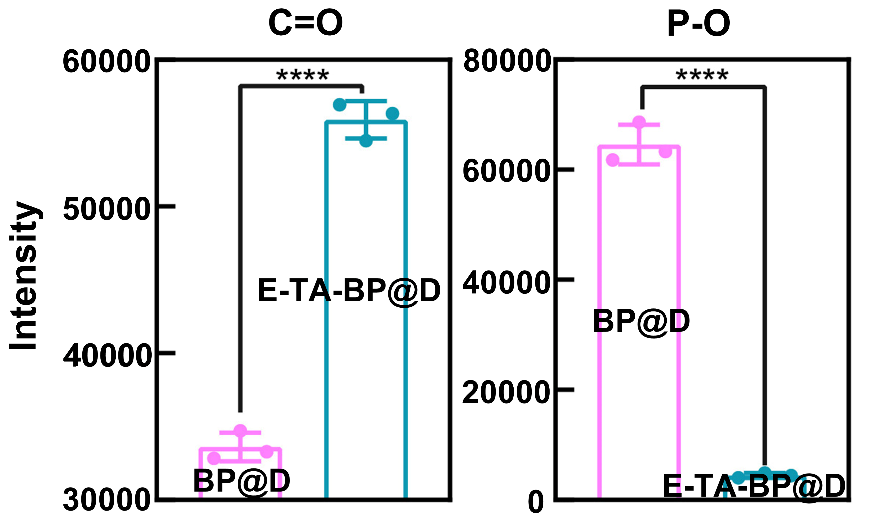


Figure S3. Characterization. Semi-quantitative analysis of C=O and P-O bonds in XPS.


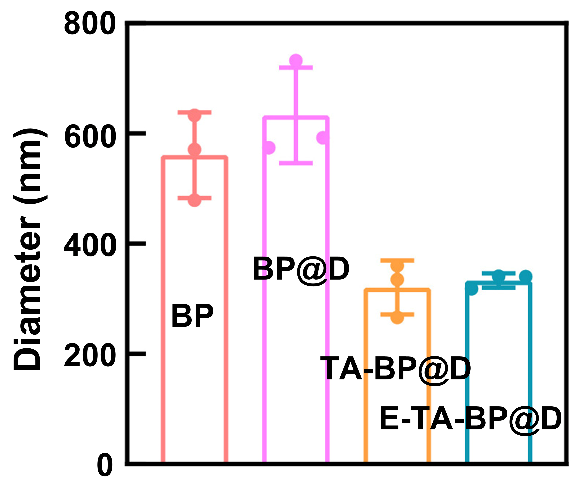


Figure S4. Characterization. Dynamic light scattering (DLS) measurement results of BP, BP@D, TA-BP@D and E-TA-BP@D.


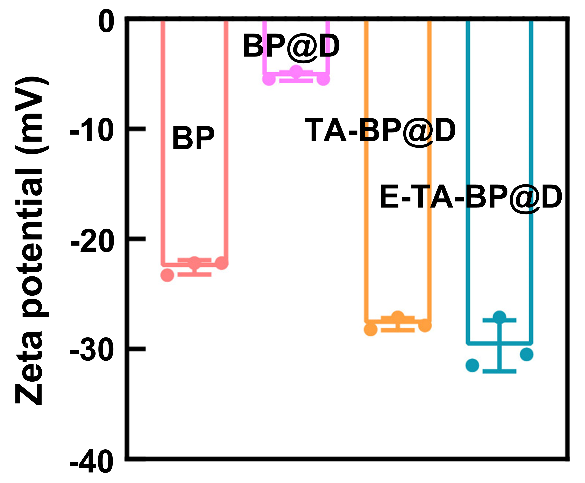


Figure S5. Characterization. Zeta potentials.


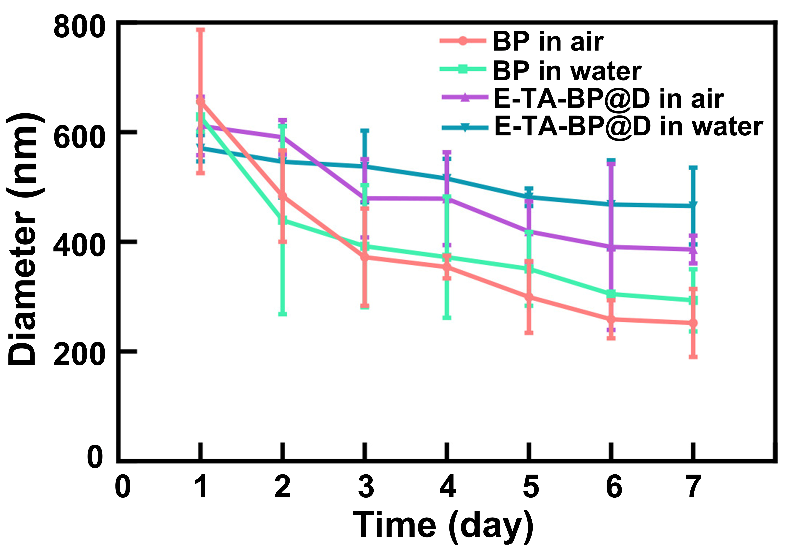


Figure S6. Characterization. DLS analysis for 7 consecutive days.


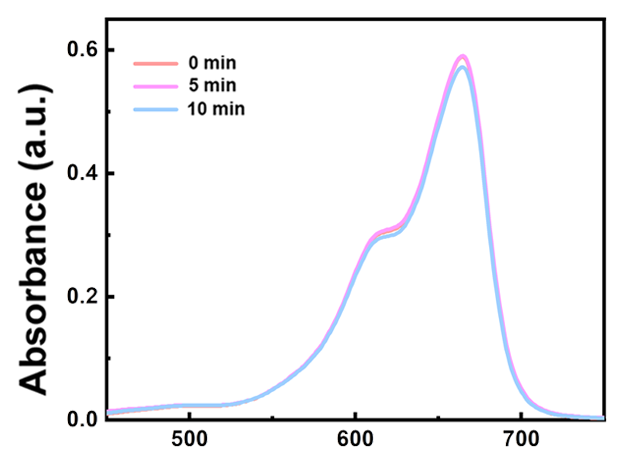


Figure S7. UV-Vis results of methylene blue (MB) control.


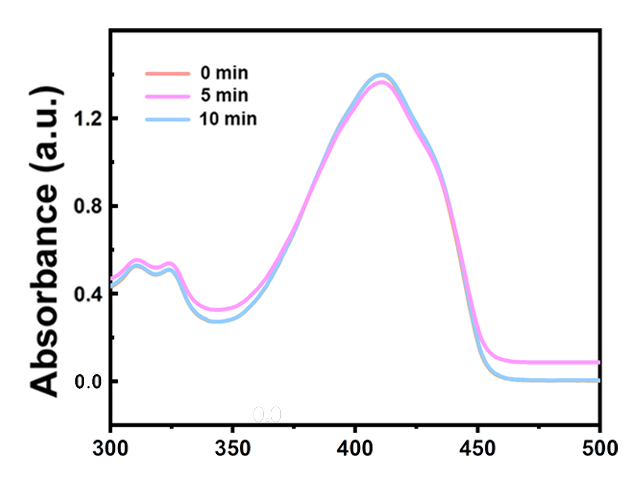


Figure S8. UV-Vis results of DPBF control.


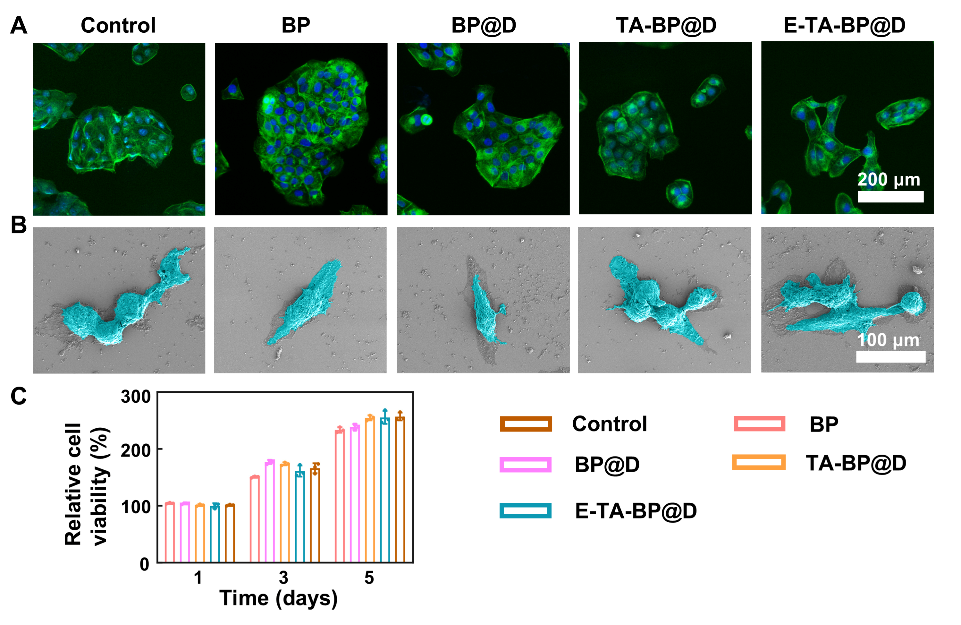


Figure S9. *In vitro* cytotoxicity of HOKs. A) Cell structure captured by CLSM. B) Morphologies of cells in different treatment groups captured by SEM. C) Cytotoxicity assessment by CCK-8 assay.


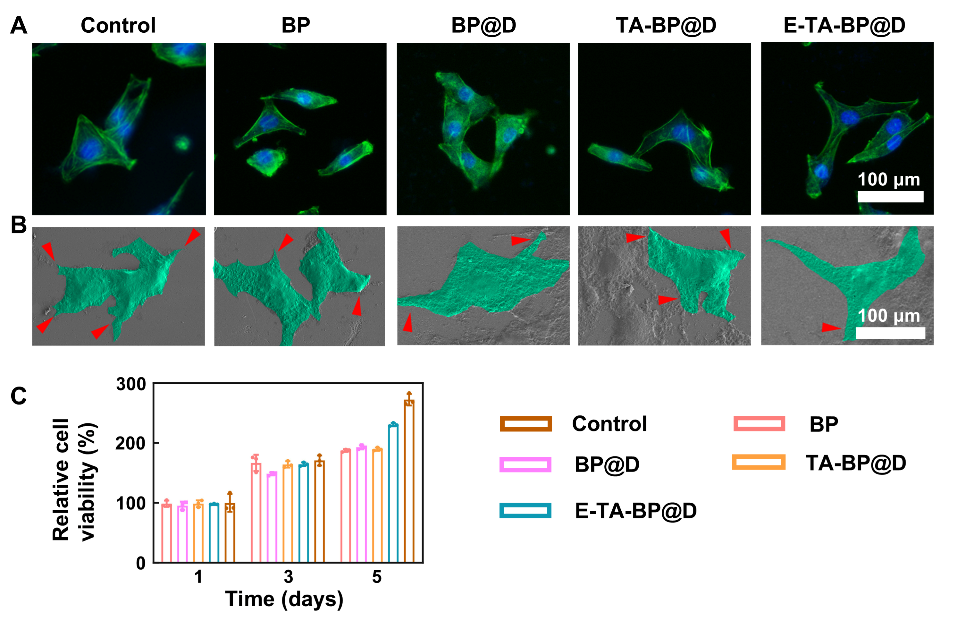


Figure S10. *In vitro* cytotoxicity of L929s. A) Cell structure captured by CLSM. B) Morphologies of cells in different treatment groups captured by SEM (the red arrows represent cellular pseudopodia). C) Cytotoxicity assessment by CCK-8 assay.


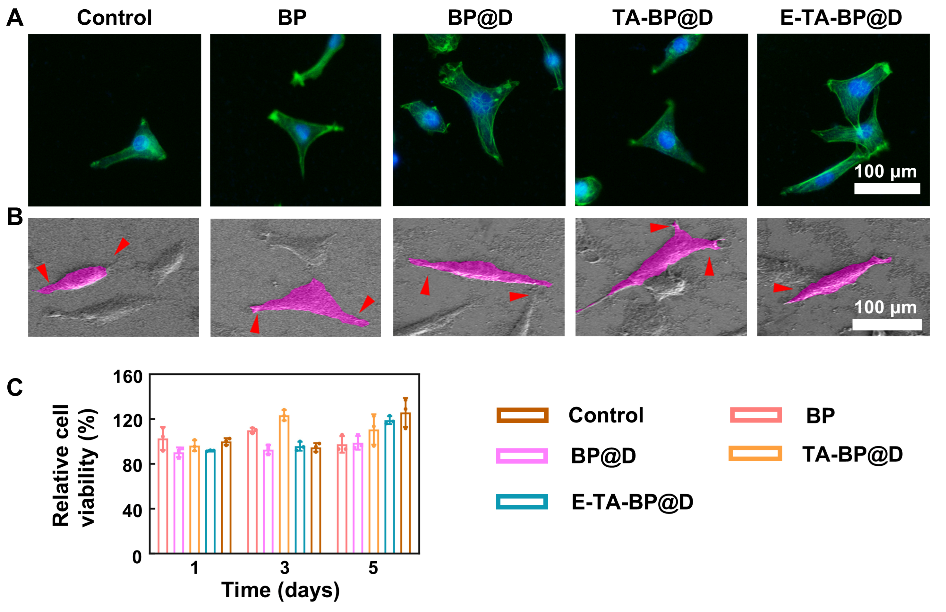


Figure S11. *In vitro* cytotoxicity of mBMSCs. A) Cell structure captured by CLSM. B) Morphologies of cells in different treatment groups captured by SEM (the red arrows represent cellular pseudopodia). C) Cytotoxicity assessment by CCK-8 assay.


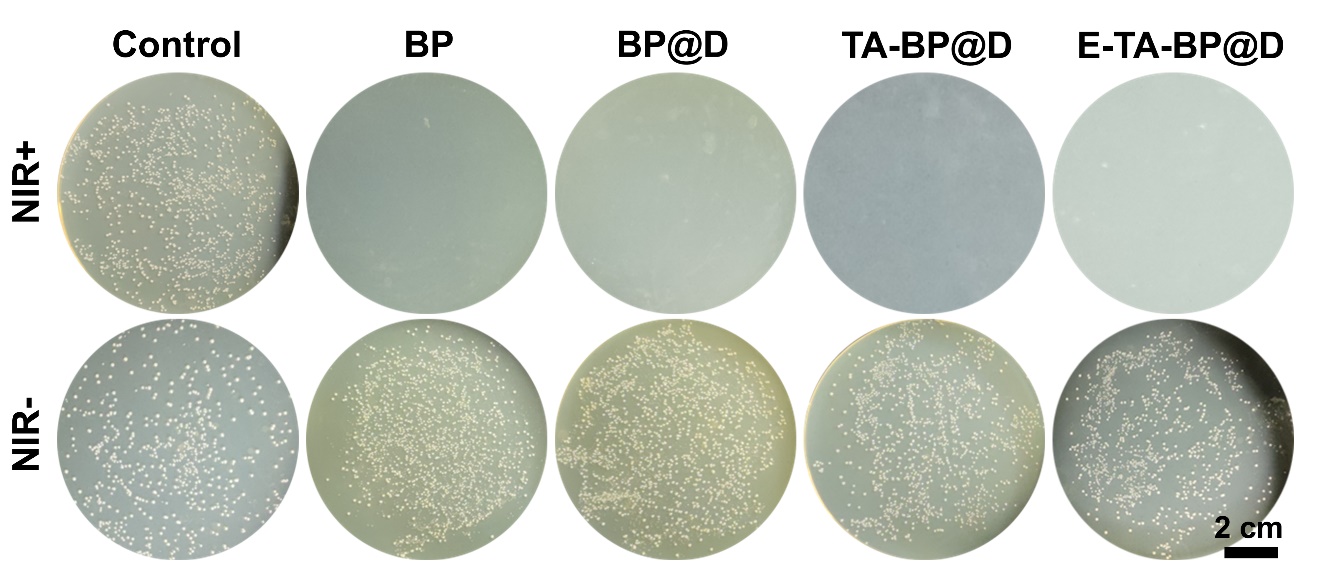


Figure S12. *In vitro* antibacterial efficiency. Typical bacterial plate images in different treatment groups (*E. faecalis*).


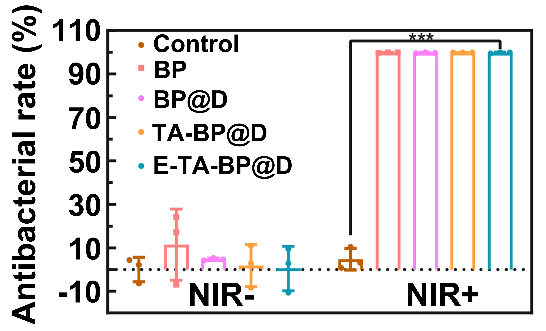


Figure S13. *In vitro* antibacterial efficiency. Antibacterial rates calculated from the plate counting results (*E. faecalis*). ***p<0.001.


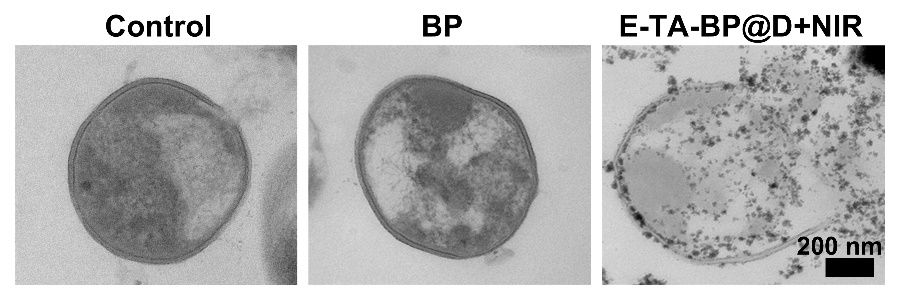


Figure S14. *In vitro* antibacterial efficiency. TEM pictures of *E. faecalis* with different treatment.


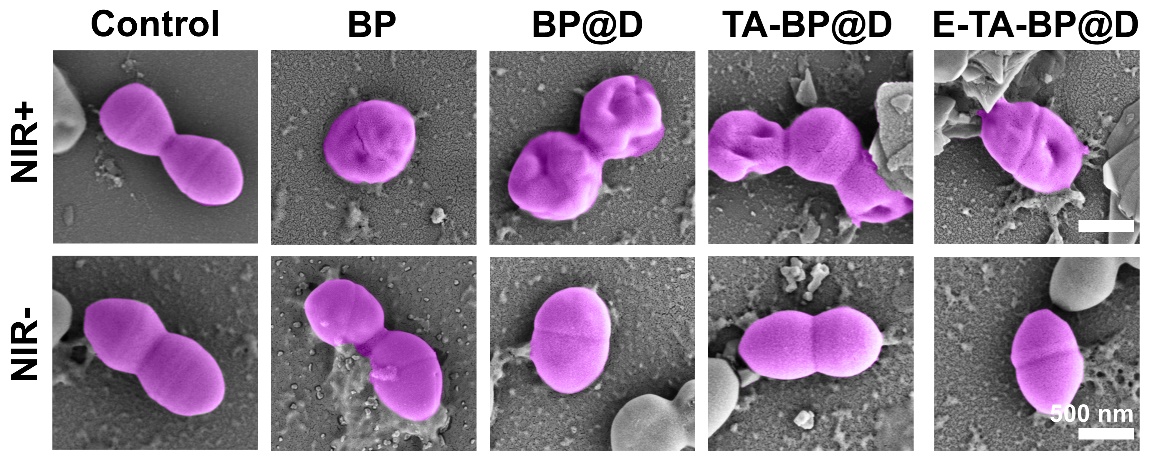


Figure S15. *In vitro* antibacterial efficiency. Morphologies of *E. faecalis* in different treatment groups captured by SEM.


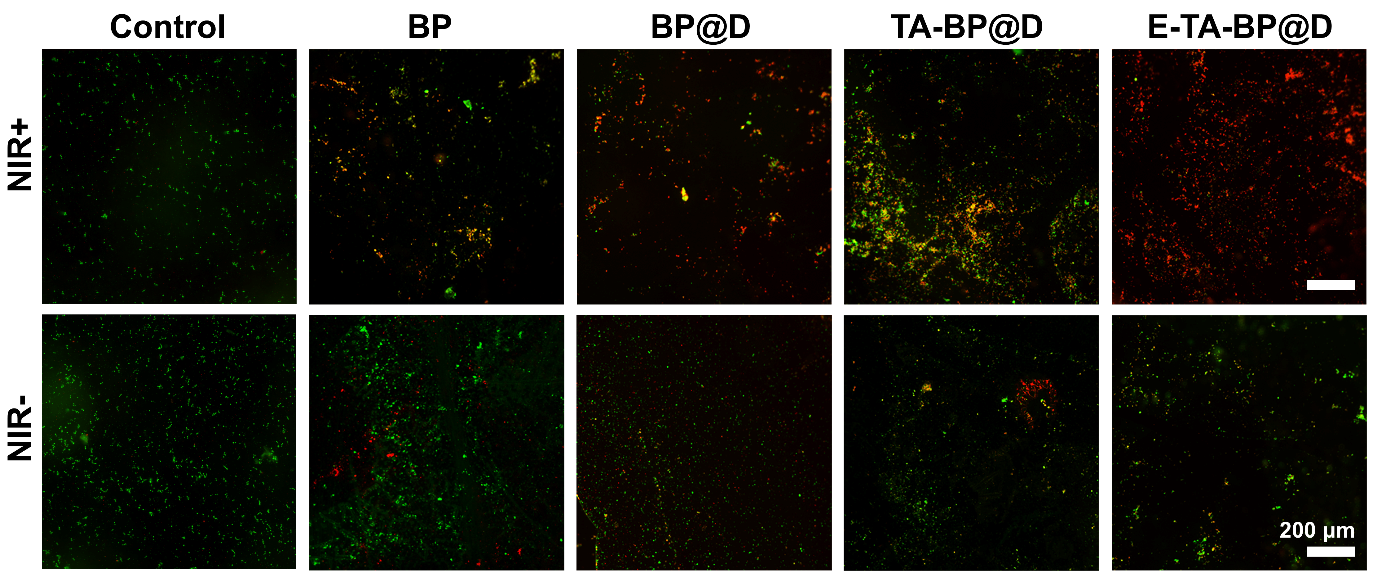


Figure S16. *In vitro* antibacterial efficiency. Representative live/dead bacterial staining images (*E. faecalis*).


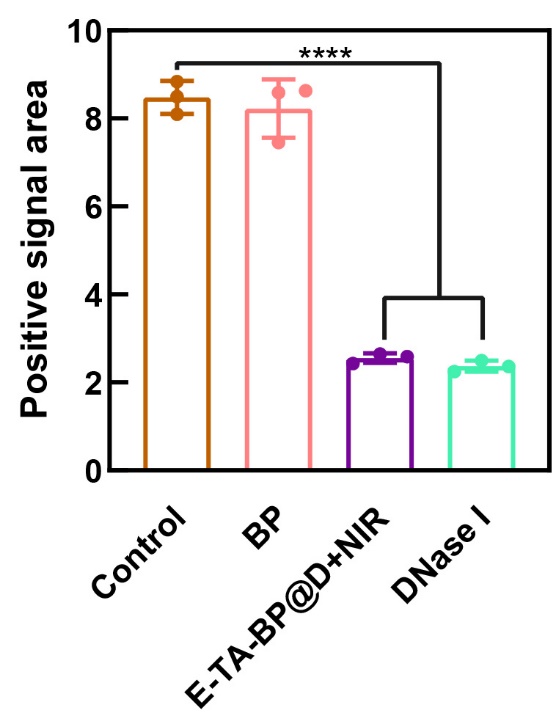


Figure S17. The quantitative analysis results of the yellow fluorescence in Figure 4A. ****p<0.0001.


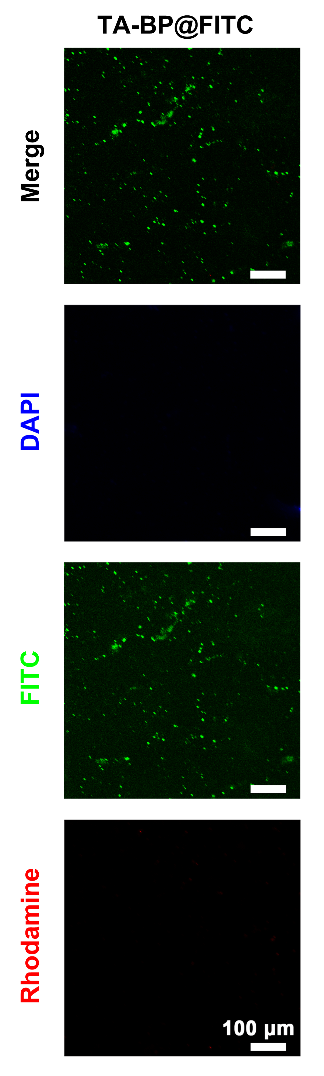


Figure S18. Fluorescence images in TA-BP@FITC group where TA-BP@FITC was present.


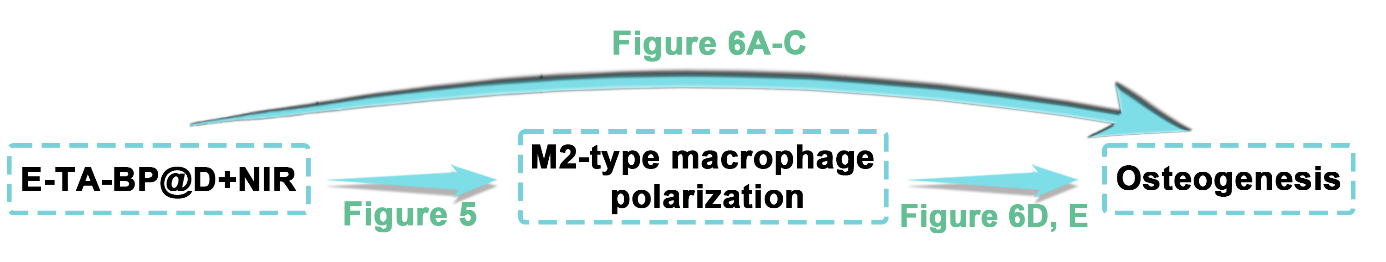


Figure S19. A flowchart illustrating the thought process leading to the conclusions in section 2.6 based on Figures 5 and 6A-E.


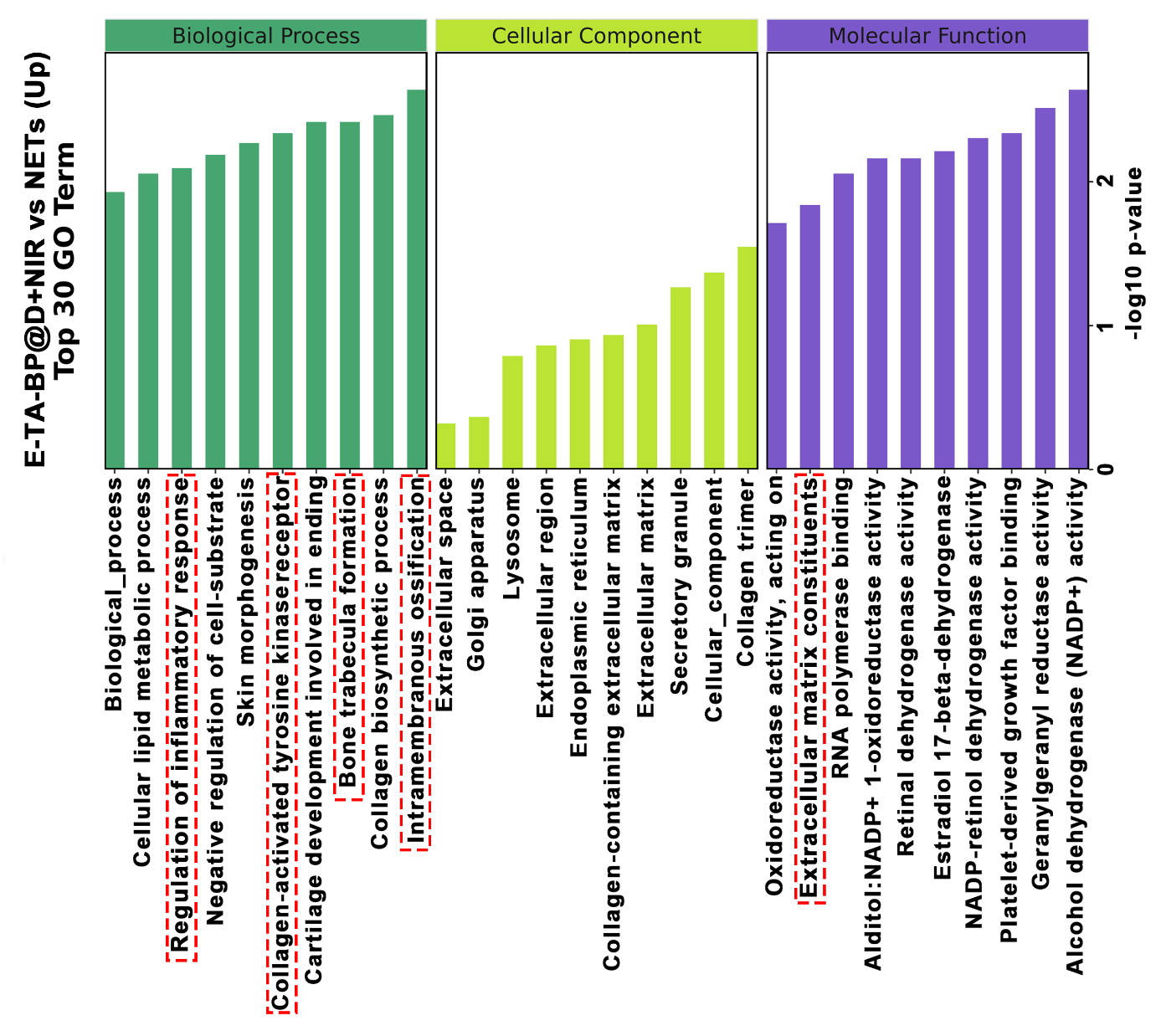


Figure S20. Gene ontology (GO) enrichment analysis of differentially expressed genes.


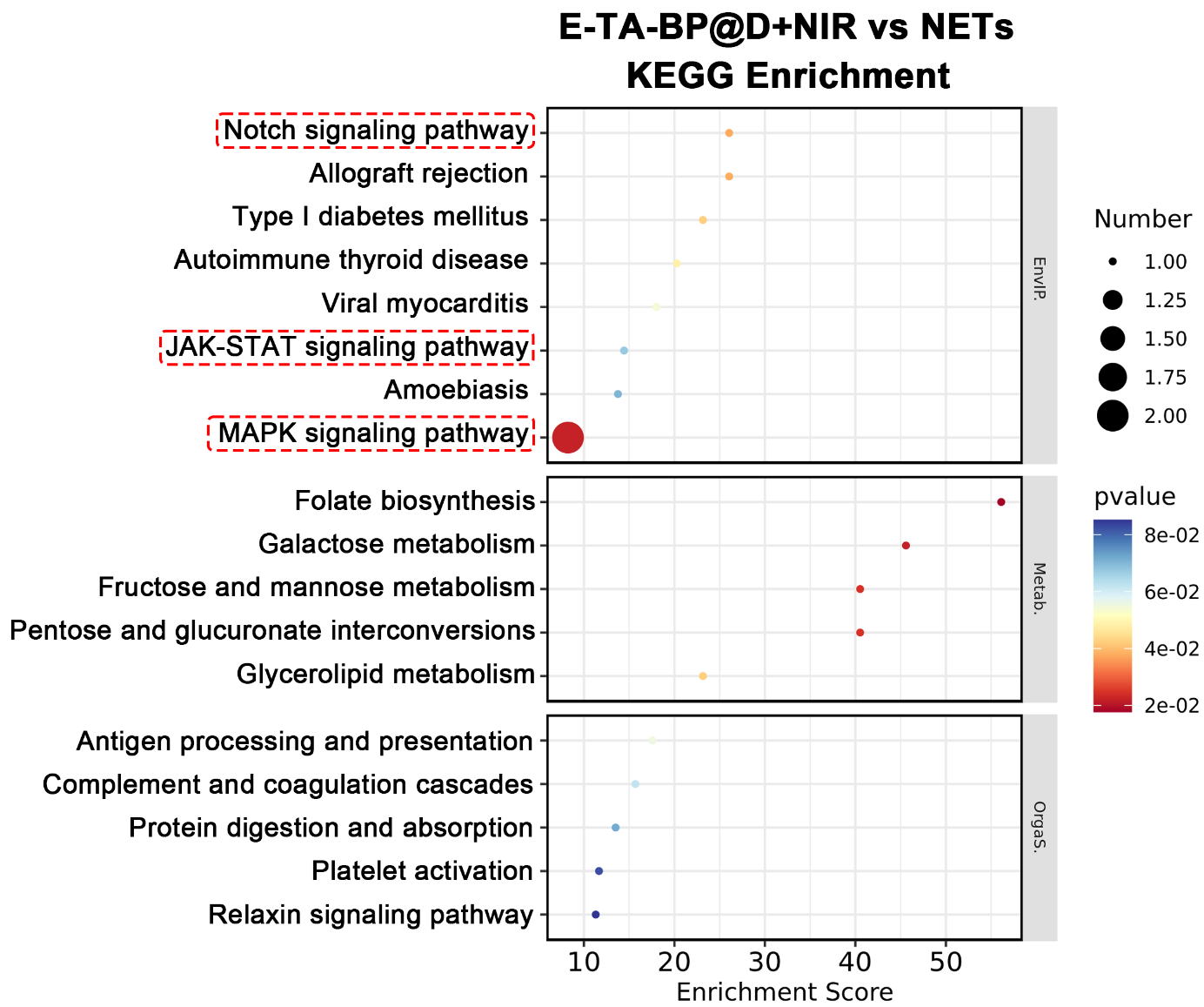


Figure S21. Kyoto Encyclopedia of genes and genomes (KEGG) analysis.


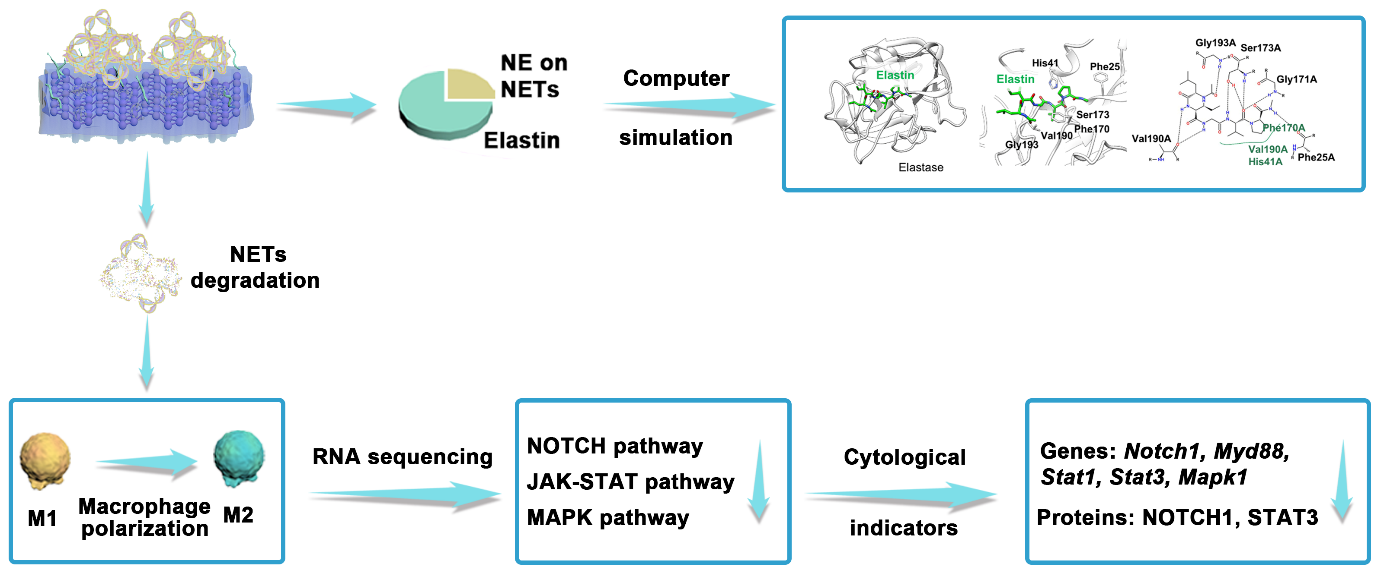


Figure S22. A schematic diagram on the mechanisms of how the nanocapsules interact with NETs. NE: neutrophil elastase.


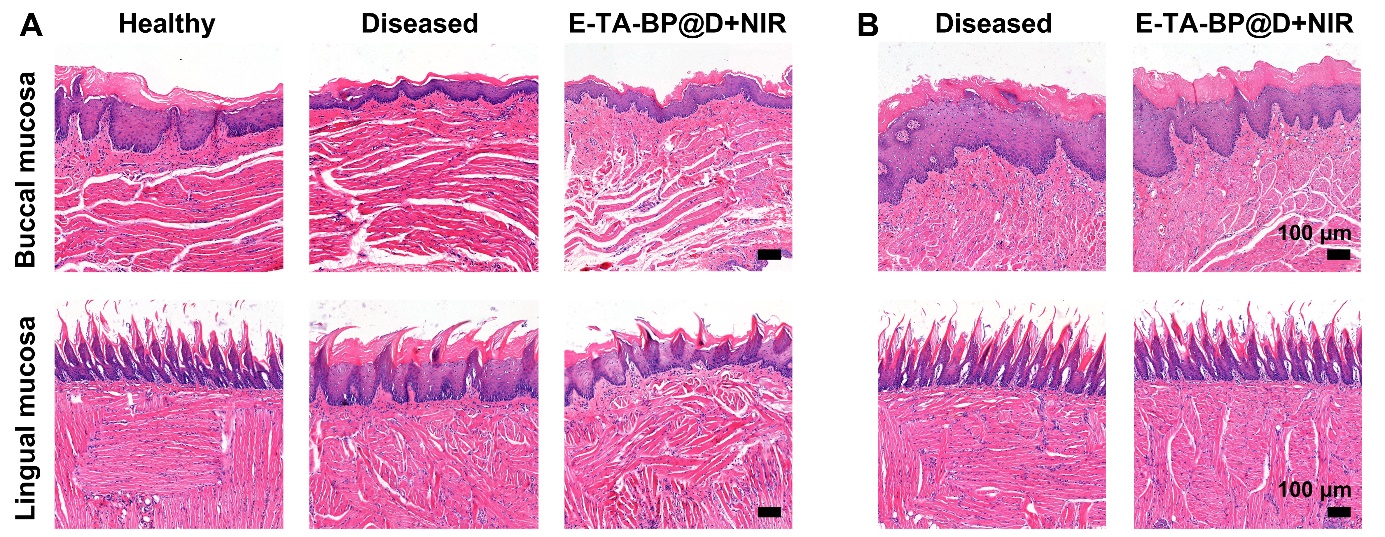


Figure S23. Hematoxylin and eosin (H&E) images for oral mucosa. A) Periodontitis model. B) Periapical periodontitis model.


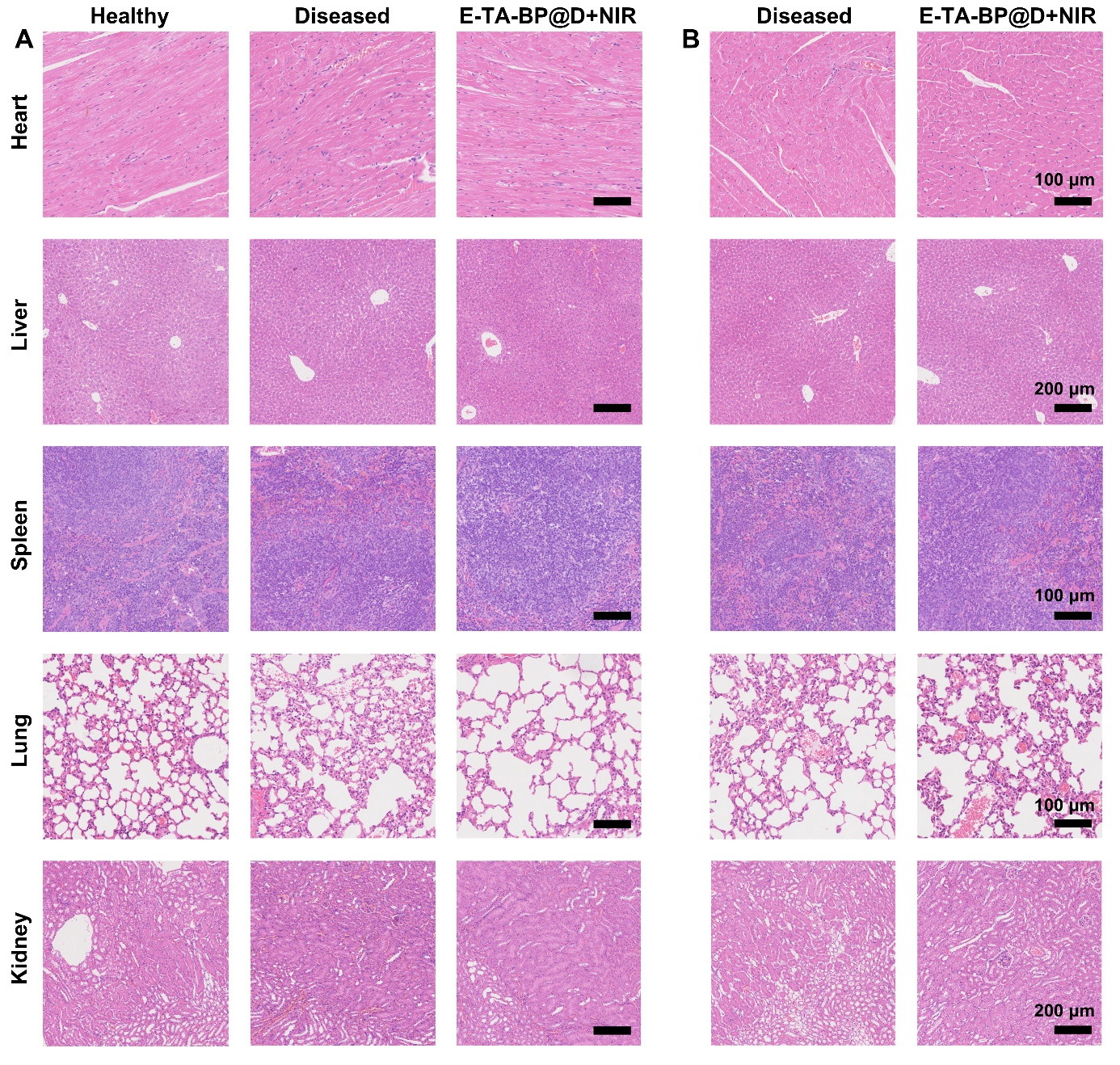


Figure S24. Hematoxylin and eosin (H&E) images for major organs. A) Periodontitis model. B) Periapical periodontitis model.


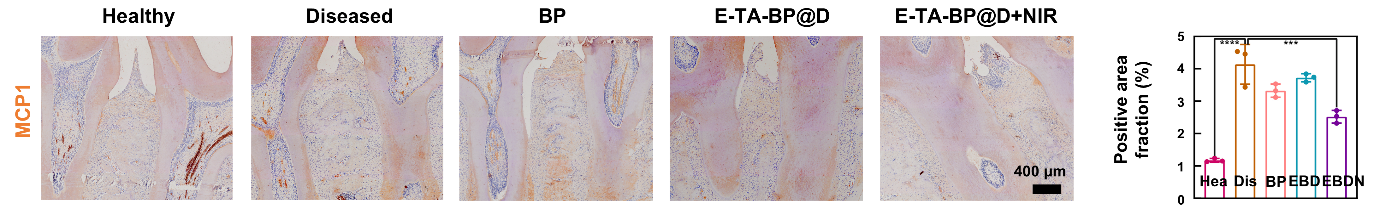


Figure S25. Neutrophil-related marker in periodontitis. Immunohistochemical staining: MCP1. ***p<0.001, ****p<0.0001. Hea: Healthy, Dis: Diseased, EBD: E-TA-BP@D, EBDN: E-TA-BP@D+NIR.


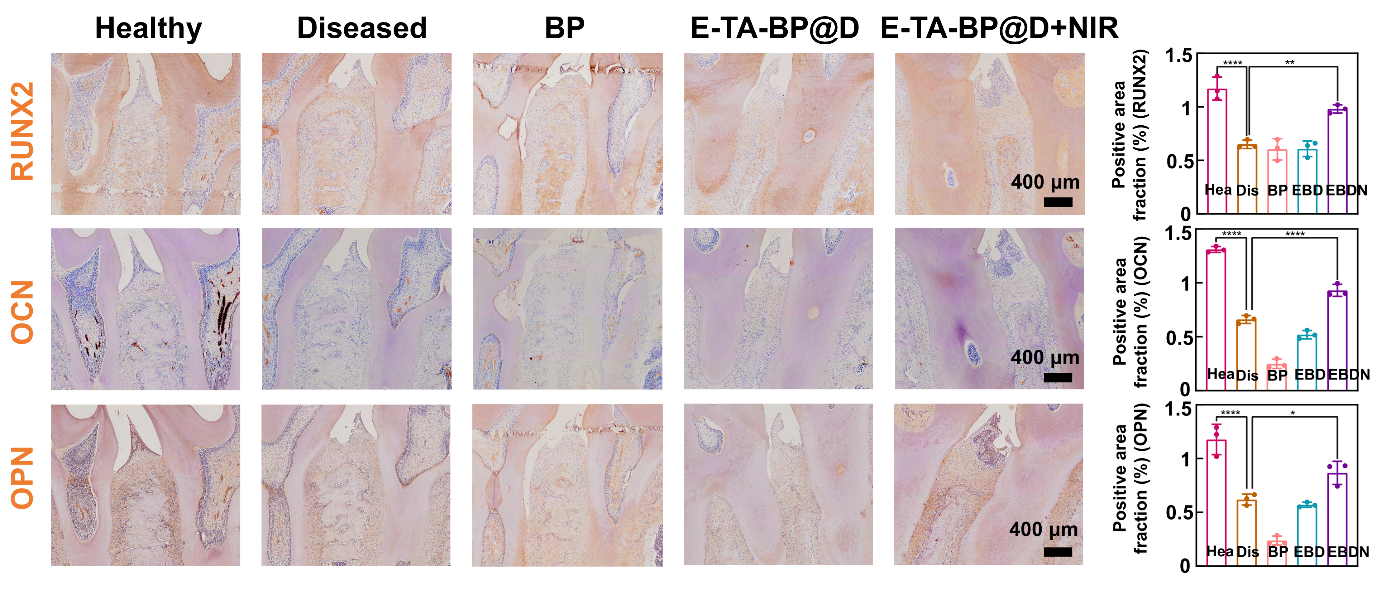


Figure S26. Expression of osteogenic-related proteins periodontitis. Immunohistochemical staining: RUNX2, OCN, OPN. *p<0.05, **p<0.01, ****p<0.0001. Hea: Healthy, Dis: Diseased, EBD: E-TA-BP@D, EBDN: E-TA-BP@D+NIR.


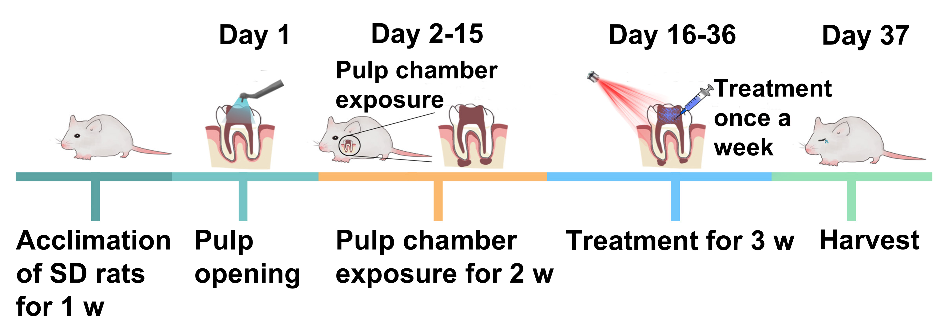


Figure S27. *In vivo* therapeutic efficiency for periapical periodontitis. Schematic diagram of the animal experiments.


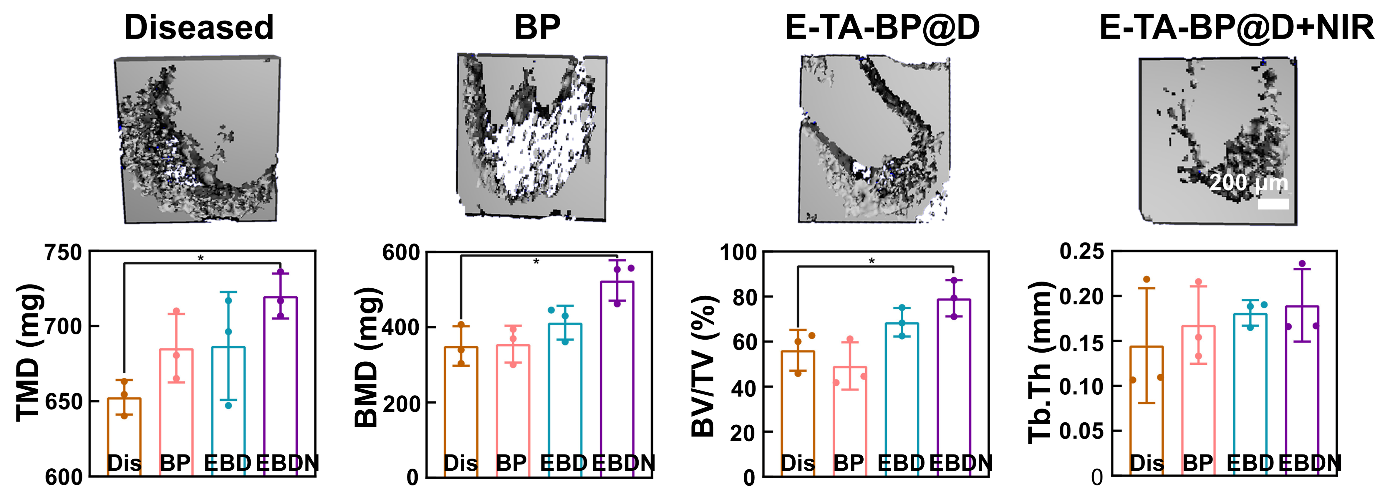


Figure S28. *In vivo* therapeutic efficiency for periapical periodontitis. The three-dimensional reconstruction sections and quantification in micro-computed tomography (micro-CT) assay. *p<0.05. Dis: Diseased, EBD: E-TA-BP@D, EBDN: E-TA-BP@D+NIR.


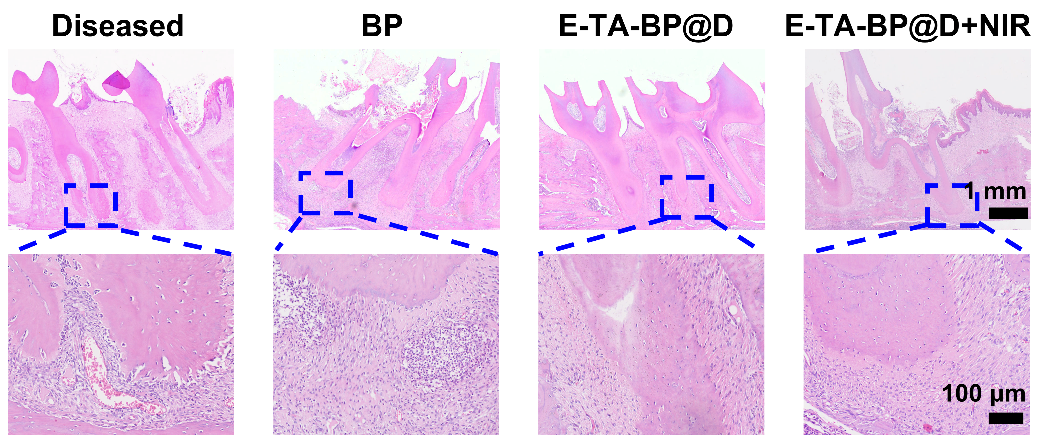


Figure S29. *In vivo* therapeutic efficiency for periapical periodontitis. H&E-stained paraffin sections.


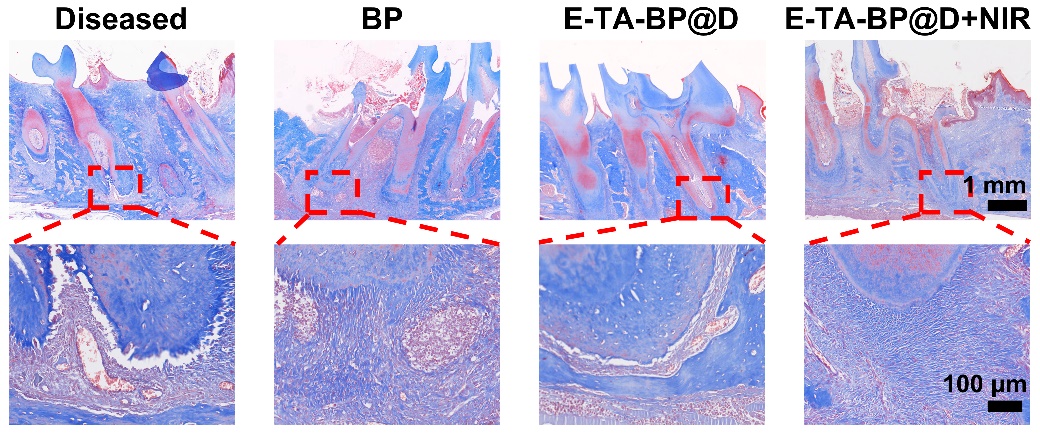


Figure S30. *In vivo* therapeutic efficiency for periapical periodontitis. Masson’s trichrome staining.


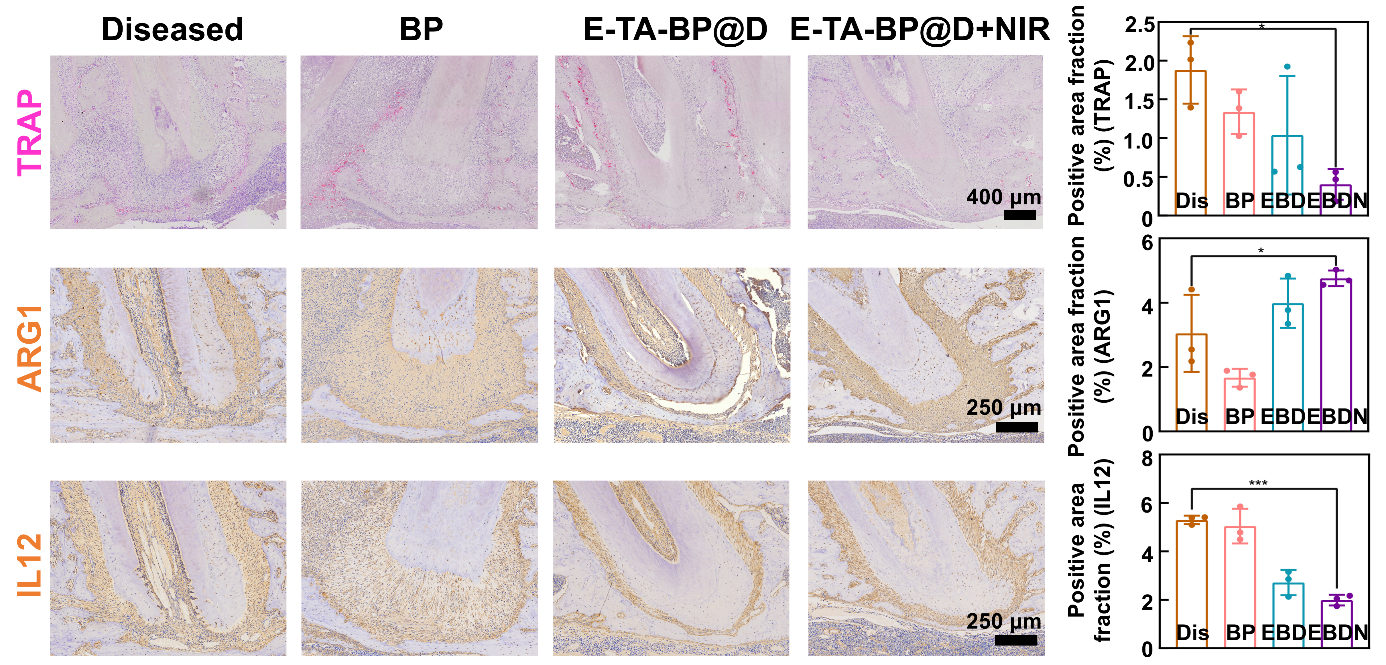


Figure S31. *In vivo* therapeutic efficiency for periapical periodontitis. Immunohistochemical staining: TRAP, ARG1, IL-12. *p<0.05, ***p<0.001. Dis: Diseased, EBD: E-TA-BP@D, EBDN: E-TA-BP@D+NIR.


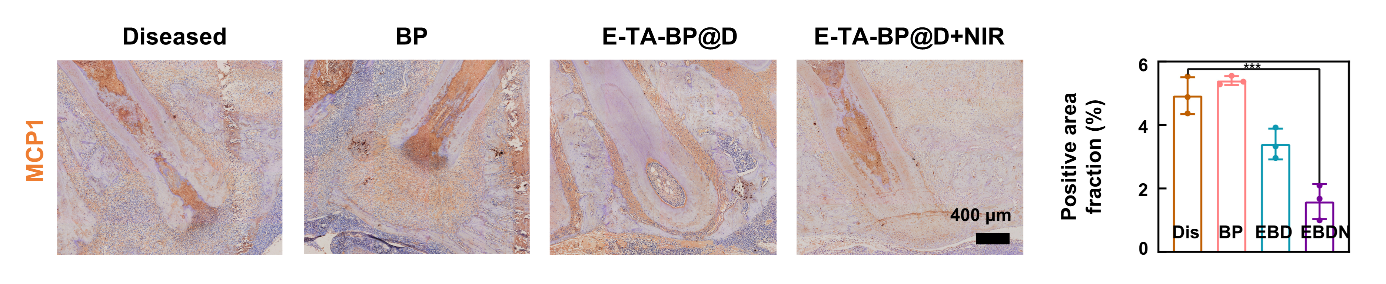


Figure S32. Neutrophil-related marker in periapical periodontitis. Immunohistochemical staining: MCP1. ***p<0.001. Dis: Diseased, EBD: E-TA-BP@D, EBDN: E-TA-BP@D+NIR.


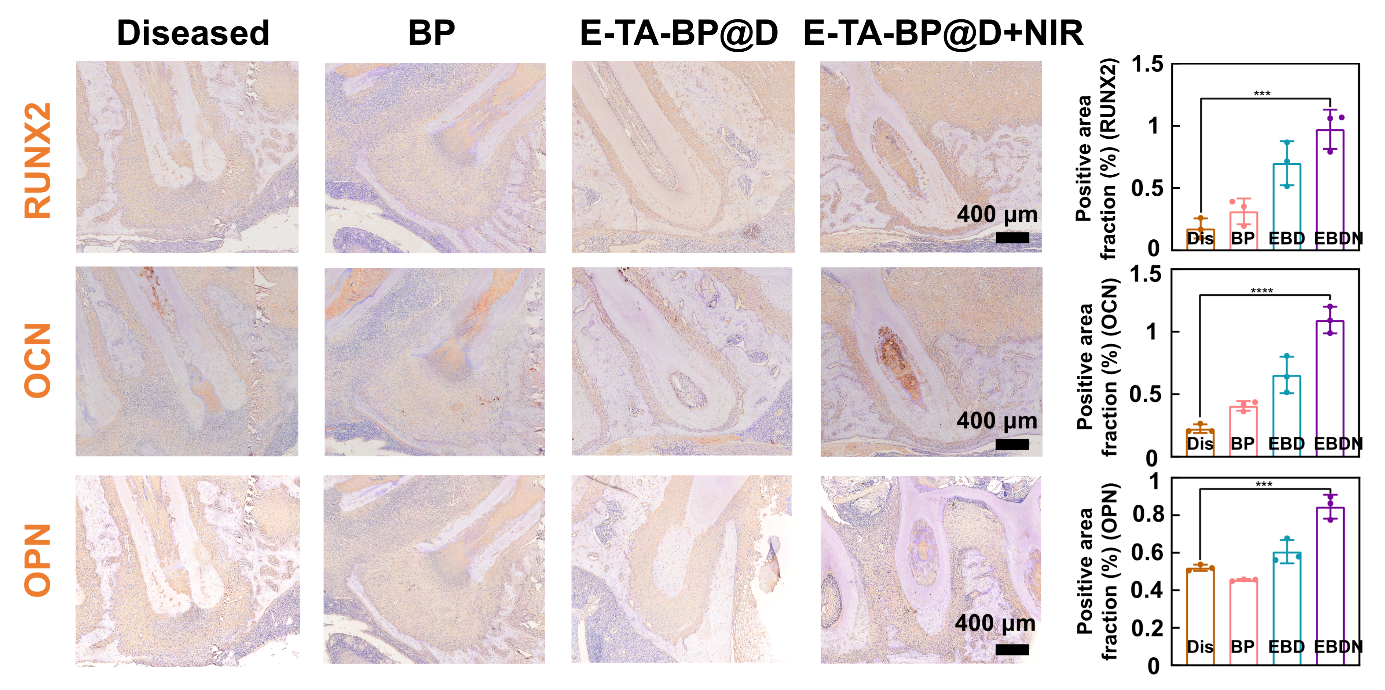


Figure S33. Expression of osteogenic-related proteins in periapical periodontitis. Immunohistochemical staining: RUNX2, OCN, OPN. ***p<0.001, ****p<0.0001. Dis: Diseased, EBD: E-TA-BP@D, EBDN: E-TA-BP@D+NIR.


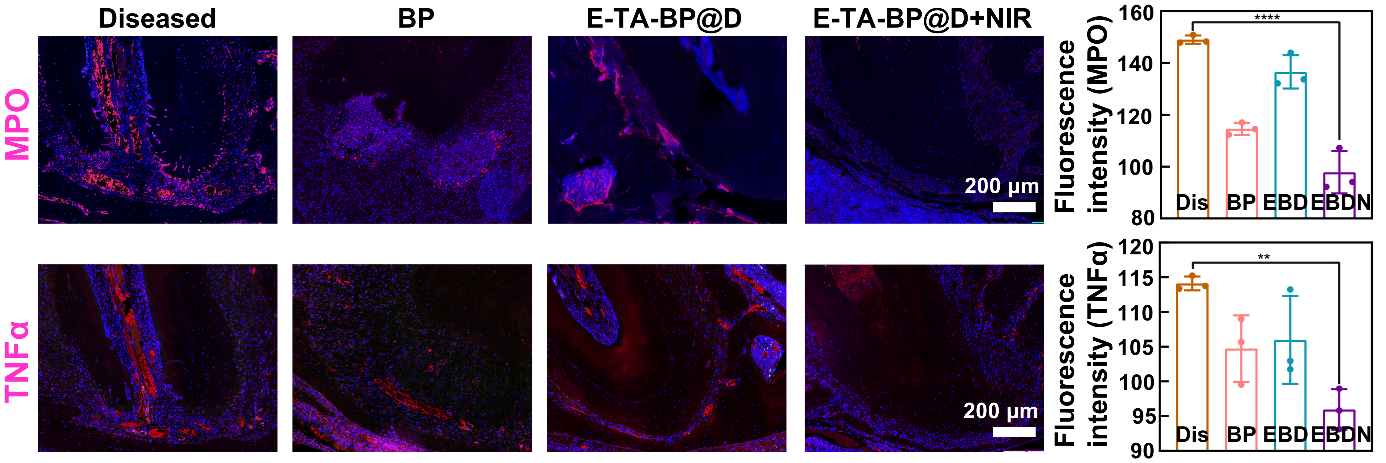


Figure S34. *In vivo* therapeutic efficiency for periapical periodontitis. Immunofluorescence staining: MPO, TNFα. **p<0.01, ****p<0.0001. Dis: Diseased, EBD: E-TA-BP@D, EBDN: E-TA-BP@D+NIR.

**Tables**

**Table S1. Calculated binding scores using different scoring functions.**

| Scoring functions | Calculated binding scores |
| --- | --- |
| Total Score | 3.98 |
| D_Score | -159.8366 |
| PMF_Score | -16.0539 |
| G_Score | -337.8839 |
| CHEMSCORE | -28.0838 |
|  | |

**Table S2. Primer sequences used in quantitative polymerase chain reaction (qPCR).**

| Gene | Sequences |
| --- | --- |
| *Gapdh* | Forward5’- ACCCTTAAGAGGGATGCTGC-3’  Reverse5’- CCCAATACGGCCAAATCCGT-3’ |
| *Cd86* | Forward5’- CAGCACGGACTTGAACAACC-3’ Reverse5’- CTCCACGGAAACAGCATCTGA-3’ |
| *Il6* | Forward5’- GACAAAGCCAGAGTCCTTCAGA-3’  Reverse5’- TGTGACTCCAGCTTATCTCTTGG-3’ |
| *Notch1* | Forward5’-CAACTGCCAGAACCTTGTGC-3’ |
|  | Reverse5’-AGAGTGACGTCAATGCCTCG-3’ |
| *Stat1* | Forward5’-GATCGCTTGCCCAACTCTTG-3’ |
|  | Reverse5’-ACTGTGACATCCTTGGGCTG-3’ |
| *Mapk1* | Forward5’-CCCAAGTGATGAGCCCATTG-3’ |
|  | Reverse5’-CTTACACCATCTCTCCCTTGCT-3’ |
| *Myd88* | Forward5’-TAGGACAAACGCCGGAACTT-3’ |
|  | Reverse5’-ATGCGGCGACACCTTTTCTC-3’ |
| *Stat3* | Forward5’-GAACCTGGGGTTCCGACG-3’ |
|  | Reverse5’-TCAGGGGTCTCGACTGTCTC-3’ |

**Table S3. Antibodies used in the study.**

| Antibody name&species | Company | Cargo number |
| --- | --- | --- |
| Rabbit Anti-CD206 | Cell Signaling Technology | 24595 |
| Rabbit Anti-iNOS | Cell Signaling Technology | 13120 |
| Rabbit Anti-GAPDH | Servicebio | GB11002 |
| Rabbit Anti-ARG1 | Servicebio | GB11285 |
| Rabbit Anti-IL12 | Servicebio | GB11113 |
| Rabbit Anti-MPO | Servicebio | GB11224 |
| Rabbit Anti-TNFα | Servicebio | GB11188 |
| Rabbit Anti -Osteopontin | Servicebio | GB115684 |
| Rabbit Anti -Osteopontin | Servicebio | GB112328 |
| Rabbit Anti-RUNX2 | Servicebio | GB115631 |
| Rabbit Anti -MCP 1 | Servicebio | GB11199 |
| Rabbit Anti -STAT3 | Servicebio | GB11176 |
| Rabbit Anti -Notch1 | Servicebio | GB111690 |
